# Mechanistic Target of Rapamycin Complex 1 Promotes the Expression of Genes Encoding Electron Transport Chain Proteins and Stimulates Oxidative Phosphorylation in Primary Human Trophoblast Cells by Regulating Mitochondrial Biogenesis

**DOI:** 10.1038/s41598-018-36265-8

**Published:** 2019-01-22

**Authors:** Fredrick J. Rosario, Madhulika B. Gupta, Leslie Myatt, Theresa L. Powell, Jeremy P. Glenn, Laura Cox, Thomas Jansson

**Affiliations:** 1 Division of Reproductive Sciences, Department of Obstetrics and Gynecology, University of Colorado Anschutz Medical Campus, Aurora, CO USA; 2 Children’s Health Research Institute and Department of Pediatrics and Biochemistry, University of Western Ontario, London, Ontario, N6A 5C1 Canada; 3 Department of Obstetrics and Gynecology, Oregon Health and Science University, Portland, USA; 4 Section of Neonatology, Department of Pediatrics, University of Colorado Anschutz Medical Campus, Aurora, CO USA; 5 Department of Genetics, Southwest National Primate Research Center, Texas Biomedical Research Institute, San Antonio, TX USA; 6 Department of Internal Medicine, Section of Molecular Medicine and Center for Precision Medicine, Wake Forest School of Medicine, Winston-Salem, NC USA

## Abstract

Trophoblast oxidative phosphorylation provides energy for active transport and protein synthesis, which are critical placental functions influencing fetal growth and long-term health. The molecular mechanisms regulating trophoblast mitochondrial oxidative phosphorylation are largely unknown. We hypothesized that mechanistic Target of Rapamycin Complex 1 (mTORC1) is a positive regulator of key genes encoding Electron Transport Chain (ETC) proteins and stimulates oxidative phosphorylation in trophoblast and that ETC protein expression is down-regulated in placentas of infants with intrauterine growth restriction (IUGR). We silenced raptor (mTORC1 inhibition), rictor (mTORC2 inhibition) or DEPTOR (mTORC1/2 activation) in cultured term primary human trophoblast (PHT) cells. mTORC1 inhibition caused a coordinated down-regulation of 18 genes encoding ETC proteins representing all ETC complexes. Inhibition of mTORC1, but not mTORC2, decreased protein expression of ETC complexes I–IV, mitochondrial basal, ATP coupled and maximal respiration, reserve capacity and proton leak, whereas activation of mTORC1 had the opposite effects. Moreover, placental protein expression of ETC complexes was decreased and positively correlated to mTOR signaling activity in IUGR. By controlling trophoblast ATP production, mTORC1 links nutrient and O_2_ availability and growth factor signaling to placental function and fetal growth. Reduced placental mTOR activity may impair mitochondrial respiration and contribute to placental insufficiency in IUGR pregnancies.

## Introduction

The placenta constitutes the primary maternal-fetal interface and its immunologic, transport, metabolic and endocrine functions are critical for normal fetal growth and development. The syncytiotrophoblast, the transporting and hormone-producing epithelium of the human placenta, requires a large amount of energy in the form of ATP to support processes such as active transport and protein synthesis. These ATP needs are met, in part, by mitochondrial respiration, however the molecular mechanisms regulating oxidative phosphorylation in primary human trophoblast (PHT) cells are largely unknown.

As in other cells, syncytiotrophoblast mitochondria are critical for a multitude of biological processes including energy metabolism, redox signaling, steroid synthesis, and apoptosis^1^. Impaired placental mitochondrial function^2^ and ATP production, placental insufficiency^3,4^, and hypoxia^3^ may act in concert to reduce fetal growth. Perturbations in trophoblast mitochondrial function could lead to excessive generation of reactive oxygen and nitrogen species^5^, contributing to altered placental function in IUGR, gestational diabetes, and maternal obesity^6–8^. Moreover, placental mitochondrial dysfunction has been implicated in the programming of atherosclerosis in cases of placental insufficiency^5^.

Mechanistic Target of Rapamycin (mTOR) is a serine/threonine kinase that is activated by amino acids, glucose, oxygen and growth factor signaling and promotes cell growth and metabolism. mTOR exists in two complexes, mTOR Complex 1 (mTORC1) and 2, with the protein Regulatory-associated protein of mTOR (Raptor) associated to mTORC1 and Rapamycin-insensitive companion of mammalian target of rapamycin (Rictor) associated to mTORC2. When activated, mTORC1 phosphorylates p70 ribosomal protein S6 kinase 1 (S6K1) and 4E-binding protein 1 (4E-BP1) promoting protein translation, lipid biogenesis and metabolism as well as suppressing autophagy^9–14^. mTORC2 phosphorylates protein kinase B (AKT), Protein kinase C alpha (PKCα) and Serum and Glucocorticoid-regulated Kinase 1 (SGK1) and regulates cytoskeletal organization and metabolism^15,16^. DEPTOR, a protein containing two DEP (Dishevelled, Egl-10, Pleckstrin) domains, is an endogenous inhibitor of both mTORC1 and 2 signaling^17^. Placental mTOR activity is decreased in human intrauterine growth restriction (IUGR)^18–20^ as well as in rodent^21^ and non-human primate models of IUGR^22^. On the other end of the fetal growth spectrum, placental mTOR activity is activated in obese women giving birth to larger babies^23^ as well as in a mouse model of maternal obesity associated with fetal overgrowth^24^. We recently demonstrated that placental mTOR functions as a positive regulator of amino acid transporter system A and L^25^ and folate transporters^26^.

mTORC1 activation increases mitochondrial DNA copy number and promotes the expression of genes critical for mitochondrial metabolism^27,28^ including genes encoding for proteins involved in oxidative phosphorylation^29,30^. Recent studies in MCF7 cells suggest that mTORC1 controls mitochondrial activity and biogenesis through 4E-BP1 dependent translation^31^. Furthermore, rapamycin-treated leukemic cells display reduced mitochondrial function, resulting in energy production via enhanced aerobic glycolysis in preference over mitochondrial respiration^31^. However, the mechanisms involved and the specific role of mTORC1 and mTORC2 signaling in the regulation of mitochondrial respiration in the placenta remain to be established.

Using gene silencing approaches in cultured primary human trophoblast cells and studies of placental tissue from normal and IUGR pregnancies we tested the hypothesis that mTORC1 is a positive regulator of key genes encoding Electron Transport Chain (ETC) proteins and stimulates oxidative phosphorylation in trophoblast and that ETC protein expression is down-regulated in placentas of infants with intrauterine growth restriction.

## Materials and Methods

### Ethical Approval and Study Participants

PHT cells were isolated from placentas of uncomplicated term pregnancies collected with informed consent at the Labor and Delivery Unit at University Hospital San Antonio with approval of the Institutional Review Board of the University of Texas Health Science Center San Antonio. Placentas from pregnancies complicated by intrauterine growth restriction (IUGR) and women delivering appropriate-for-gestational age (AGA) infants were collected at St. Joseph’s Health Care Centre, London, Ontario, Canada. These women were enrolled after informed consent was obtained, according to a protocol approved by the University of Western Ontario Health Sciences Research Ethics Board. The recruitment procedure, including inclusion and exclusion criteria, has been described in detail previously^20^. All experimental protocols and methods were approved by a University of Texas Health Science Center San Antonio committee and St. Joseph’s Health Care Centre, London, Ontario, Canada. The informed consent was obtained from all subjects.

## Materials

Oligomycin, FCCP [4-(trifluoromethoxy) phenylhydrazone], and antimycin A were obtained from Sigma and dissolved in DMSO as 2.5 mM stock solutions. A human oxidative phosphorylation (OXPHOS) antibody cocktail and antibodies targeting NDUFB8, TOM20, Cytochrome b and c antibodies were purchased from Abcam. Raptor and rictor antibodies were obtained from Cell Signaling Technology.

### Isolation and Culture of Primary human Trophoblasts (PHT)

Placentas were collected immediately following delivery by cesarean section at term without labor. PHT cells were isolated and cultured *in vitro* using a well-established protocols^26,32^. Cells were plated in either 60 mm culture dishes (~7.5 × 10^6^ cells/dish for RNA isolation or Western blot analysis) or in Seahorse XF-24 plates for mitochondrial respiration experiments (~8 × 10^5^ cells/well for RNAi mediated gene silencing) and cultured in 5% CO_2_, 95% atmosphere air at 37 °C for 90 h. Cell culture media (DMEM/Hams F-12, supplemented with L-glutamine, penicillin, streptomycin, gentamycin, and 10% fetal bovine serum) was changed daily. The study design, including the time point of siRNA mediated silencing/activation of mTORC1/C2 signaling and measurements of various parameters, is illustrated in Supplemental Fig. 1.

Mononuclear cytotrophoblast cells isolated from term placentas typically start to aggregate into monolayers of mononucleated cells within about 12 h of plating. By 72 h in culture, most cytotrophoblast cells have differentiated into multinuclear syncytiotrophoblast as evidenced by the expression of cytokeratin-7, a trophoblast specific marker, and secretion of human chorionic gonadotropin-β (β-hCG), produced exclusively by syncytiotrophoblast^33,34^. Furthermore, we recently reported that in our cultured PHT cells expression of syncytin, a marker of syncytialization cells increased over the culture period confirming syncytialization of our cell population^35^. Cell viability and degree of syncytialization are maintained to beyond 90 hours in culture^25,36^ allowing investigators to study syncytiotrophoblast function between 72 and 90 h. Studies at 90 h in culture ensure that maximum gene silencing has been achieved following siRNA transfection at 18 hours in culture, as in the current report.

### RNA interference-mediated silencing

Dharmafect 2 transfection reagent (Thermo Scientific, Rockford, IL) and small interfering RNAs (siRNAs) (Sigma-Aldrich, St. Louis, MO), targeting raptor (100 nM; sense, 5′CAGUUCACCGCCAUCUACA), rictor (100 nM; sense, 5′CGAUCAUGGGCAGGUAUUA), or DEPTOR (SASI_1297010-H/5582, 1297011-H) were used. Control cells were transfected with a non-coding scrambled sequence (100 nM; sense: 5′GAUCAUACGUGCGAUCAGATT). siRNA were added to cultured primary trophoblast cells (~8 × 10^5^ cells/well in 24 well plate; ~7.5 × 10^6^ cells in 60 mm dish) after 18 h in culture, incubated for 24 h, and removed, and fresh medium was added to wells^37^. At 90 hours in culture, efficiency of target silencing was determined at the protein level (expression of raptor, rictor or DEPTOR) using Western blot.

### RNA isolation from PHT cells

RNA was isolated from cultured PHT cells at 90 hr in culture using TRIzol Reagent (Invitrogen, Carlsbad, CA) according to the manufacturer’s instructions. RNA was resuspended in 100 µl DEPC-treated water. RNA quality was determined using an Agilent 2100 Bioanalyzer (Agilent Technologies, Inc., Santa Clara, CA) and RNA concentrations confirmed by quantitation using a NanoDrop™ 8000 spectrophotometer (Thermo Fisher Scientific, Wilmington, DE).

### Gene expression profiling in PHT cells

Whole genome expression profiling was performed using gene arrays (HumanHT-12 v4 Expression BeadChips, Illumina Inc., San Diego, CA). cRNA was synthesized and biotin labeled (cat. no. 1750, Ambion, Austin, TX) according to manufacturer’s instructions. Total RNA was used for first and second strand cDNA synthesis followed by *in vitro* transcription to synthesize biotin-labeled cRNA. Following quality check, cRNA was hybridized to Human HT-12 v4 Expression BeadChips (Illumina Inc.). Individual cRNA samples were used to interrogate each BeadChip (Scramble siRNA for raptor, n = 4; Raptor siRNA, n = 4; Scramble siRNA for rictor, n = 4; Rictor siRNA, n = 4). Gene expression was detected and cleaned using GenomeStudio software (Illumina Inc.) and filtered using quality score (>0.95). Gene array data were all-median normalized and log_2_ transformed (GeneSifter), and differentially expressed genes were identified by t-test (p < 0.05). Genes significantly different in expression were overlaid onto Kyoto Encyclopedia of Genes and Genomes (KEGG) pathways^38–40^ using GeneSifter. Z-scores were calculated in GeneSifter. Statistical enrichment of differentially expressed genes in pathways was determined by calculating z-scores using the following formula: z-score = (r − n(R/N))/SQRT(n(R/N) (1 − (R/N))(1 − (n − 1/N − 1)); where R = total number of genes meeting selection criteria, N = total number of genes measured, r = number of genes meeting selection criteria with the specified GO term, and n = total number of genes measured with the specific GO term. Pathway enrichment analysis of differentially expressed genes has been shown to reveal pathways that are impacted in that biological system^41^. KEGG is a comprehensive knowledge base for assisting in the biological interpretation of large-scale molecular datasets. Using KEGG pathway enrichment analysis provides a means to statistically filter gene expression data within the framework of annotated biological systems^38^. Calculations were performed using GeneSifter software default settings without user input. Pathways were considered significantly different between groups if the z-score for that pathway was greater than +2.0 or less than −2.0^42^. Biological pathways were mapped by using online tool KEGG (http://www.genome.jp/kegg/; Kyoto encyclopedia for genes and genomes)^38–40,43^.

### Western blotting

Cells were rinsed once with ice-cold PBS and lysed in ice-cold buffer (PBS containing 0.05% SDS and protease, phosphatase inhibitor). Subsequently, cells were scraped, collected, and sonicated. The soluble fraction of cell lysates were isolated by centrifugation at 13,000 g for 10 min at 4 °C. Total protein concentration in the cell lysate was determined using Bradford’s reagent (Bio-Rad). Cell lysate proteins (10 μg) were separated on 4–20% precast linear gradient gels (Invitrogen). Membranes were incubated overnight at 4 °C with primary antibody diluted in 1% nonfat milk (wt/vol) in TBST and detected using an appropriate peroxidase-conjugated secondary antibody. Products were visualized by ECL chemiluminescence (Millipore). Band intensities were measured using the G-box system (Syngene). Anti-β actin was from Sigma-Aldrich, St. Louis, Mo. Target band densities were normalized to loading using beta-actin. For each protein target the mean density of the control sample bands was assigned an arbitrary value of 1. All individual densitometry values were expressed relative to this mean.

### Assessment of Mitochondrial Function

Mitochondrial function in PHT cells was measured using a Seahorse XF24 analyzer (Seahorse Biosciences) as described^44^. The experimental details explaining the measurement of Oxygen Consumption Rate (OCR) in PHT cells is provided as Supplemental Material. Basal respiration was calculated from four baseline OCR readings. ATP coupled, maximum respiration, reserve capacity, proton leak, and non-mitochondrial respiration were calculated from OCR readings following the injection of oligomycin (1 μM), FCCP (1 μM), and antimycin A (1.5 μM).

### PHT cell ATP Levels

ATP levels were determined in cell lysates at 90 hr of culture using the Enliten ATP assay (Promega) according to the manufacturer’s protocol.

### Mitochondrial Biogenesis

#### Mitochondrial DNA copy number

At 90 hr of culture total genomic DNA was isolated from cultured PHT cells using the GenElute Mammalian Genomic DNA Miniprep Kit (Sigma-Aldrich). Mitochondrial DNA copy number was determined using the Nuvaquant human mito/nuclear DNA ratio kit (Calbiochem) involving real-time PCR and using primers for mitochondrial 16S rRNA and nuclear β2-microglobulin according to the manufacturer’s protocol.

#### Citrate synthase activity

Was measured in PHT cell lysates at 90 hr of culture using a commercial kit from Detroit R&D according to the manufacturer’s protocol. Cultured PHT cells were homogenized using lysis reagent (C3228; Sigma).

#### Staining of mitochondria in PHT cells

Isolated PHT cells (500,000 cells/well) were grown on chamber slides (Lab-Tek) for 90 hours. At 90 hr, cells were incubated with mito green indicator (Abcam) in a 37 °C, 5% CO_2_ for 10 minutes. Subsequently, cells were fixed in paraformaldehyde (4%) for 20 min. Confocal microscopy was performed using Zeiss LSM 780 microscope at 63x magnification using oil immersion. Images were captured in the same laser settings with four Z-step of 0.4 um. For densitometric analysis of mitochondrial staining, Image J Software (version 1.52a) was used. In each section, at least five randomly selected microscopic fields were used to calculate mitochondria staining density per mm^2^ and data were averaged to represent a single placenta.

### Placental mTORC1 signaling and expression of mitochondrial electron transport complexes in IUGR

Placentas from pregnancies complicated by intrauterine growth restriction (IUGR) and women delivering appropriate-for-gestational age (AGA) infants were collected within 15 minutes of delivery as described elsewhere^20^. Selected clinical data for the control and IUGR groups are listed in Table 1. There was no significant difference in maternal age, body mass index (BMI) or gestational age between the control and the IUGR groups. Birth weight was 28% lower (P < 0.01) and placental weight was reduced by 36% (P < 0.001) in the IUGR group compared with controls. The decidua basalis and chorionic plate were removed and villous tissue was dissected and rinsed in cold physiological saline. The villous tissue was transferred to cold buffer D (250 mM sucrose, 10 mM HEPES, pH 7.4) containing 1:100 dilution of protease and phosphatase inhibitors (Sigma-Aldrich, St. Louis, MO) and homogenized on ice with a Polytron (Kinematica, Luzern, Switzerland). Placental homogenates were frozen in liquid nitrogen and stored at −80 °C until further processing. The phosphorylation of key proteins in the mTORC1 [as reported in^20^], raptor, rictor, DEPTOR and mitochondrial electron transport complexes expression in placental homogenates of IUGR and control group were determined using Western blots as described above for cells.

**Table 1:** Selected clinical data.

	Control (n = 19)	IUGR (n = 25)
Maternal age (years)	25.9 ± 1.29	28.7 ± 1.23
BMI (kg/m^2^)*	28.3 ± 2.6	26.8 ± 2.0
Gestational age (weeks)	33.9 ± 0.95	35.7 ± 0.61
Birth weight (g)	2493 ± 236	1804 ± 110^†^
Birth weight percentile^‡^	55.9 ± 4.6	2.4 ± 0.3^§^
Placental weight (g)	566 ± 42.0	394 ± 18.4^||^
Fetal sex (M/F)	7/12	8/17
Mode of delivery (C/V)	6/13	15/10

Data are presented as means ± S.E.M. Abbreviations: C, caesarean section; F, female; M, male; n, numbers; V, vaginal delivery. *n = 10 (control) and 18 (IUGR); ^‡^by corresponding gestational age; ^†^*P* < 0.05; ^||^*P* < 0.01; ^§^*P* < 0.0001.

### Data presentation and statistics

The number of experiments (n) represents the number of placentas studied. Each condition was studied in triplicate, and data were averaged to represent trophoblast cells isolated from one placenta. Data are presented as means ± S.E.M or + S.E.M. Array data from each sample were all-median normalized and log_2_ transformed. Statistical analyses of array data were performed by t-test using GeneSifter software (Geospiza, Inc.) for pairwise comparisons (GeneSifter.Net, VizX Labs, Seattle, WA; GEO accession number: GSE40878). Statistical significance of differences between control and experimental groups in studies of protein expression and mitochondrial function was assessed using Student’s t-test. A P-value < 0.05 was considered significant.

## Results

### Validation of silencing efficiency

To confirm that the siRNA reduced expression of the proteins encoded by the targeted genes, we performed western blots on siRNA-transfected PHT cells at 90 h of culture (Supplemental Fig. 2). Raptor or rictor siRNA silencing in PHT cells (Supplemental Fig. 2; n = 4/each group) markedly decreased the protein expression of raptor and rictor, respectively.

### Inhibition of mTORC1 caused a decrease in expression of genes involved in oxidative phosphorylation

In a separate unpublished study we used whole genome expression profiling in raptor (mTORC1 inhibition) and rictor (mTORC2 inhibition) silenced PHT cells as an approach to identify novel trophoblast functions regulated by mTOR signaling. Leveraging this discovery data set we specifically asked the question if trophoblast mTORC1 regulates the transcription of genes encoding ETC proteins, in analogy with other cell types^27,45,46^. Indeed, expression of 18 genes encoding ETC proteins representing all five complexes involved in mitochondrial respiration was all down-regulated in response to raptor silencing (Supplemental Fig. 3), but not following rictor silencing (data not shown). In agreement with these findings, mitochondrial oxidative phosphorylation was one of the top ranked down-regulated pathways following mTORC1 inhibition using the Kyoto Encyclopedia of Genes and Genomes (KEGG; Supplemental Fig. 3).

### Effect of mTORC1 or mTORC2 inhibition on protein expression of electron transport chain complexes

**Figure 1:**
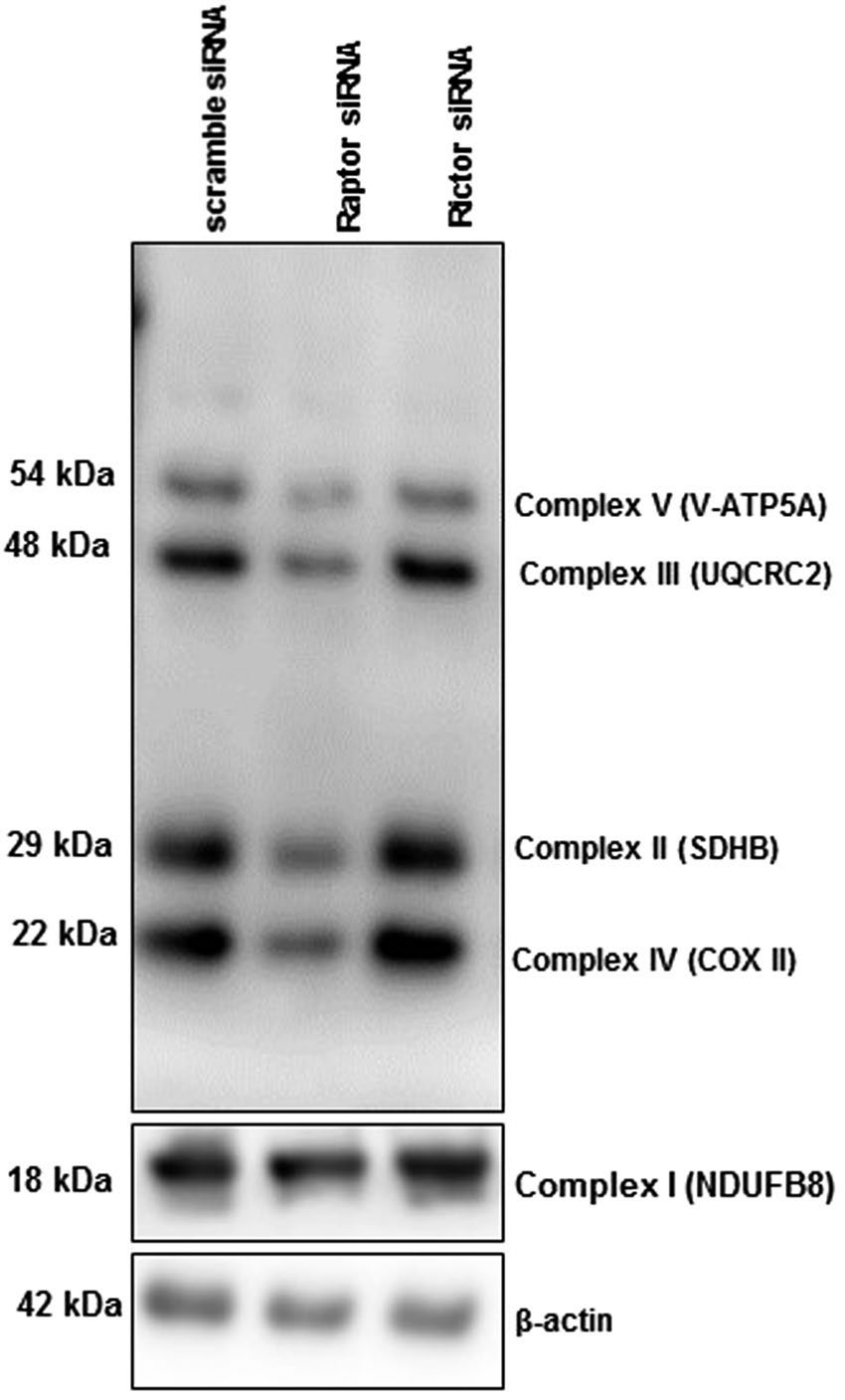
Expression of mitochondrial electron transport chain (ETC) proteins in PHT cells with mTORC1 or mTORC2 inhibition. Protein expression of ETC complex I (NDUFB8), complex II (SDHB), III (UQCRC2), IV (COXII) and V (V-ATP5A in PHT cells transfected with either scramble (n = 4) or raptor (mTORC1 inhibition, n = 4) or rictor (mTORC2 inhibition, n = 4) siRNA. Full length Western blot is shown. The data are from a representative experiment, and similar results were obtained from three other experiments.

Energy derived from the oxidation of carbohydrates, fats and proteins are transformed to ATP by the mitochondrial respiratory chain which is composed of five separate protein complexes (I–V) present in the inner mitochondrial membrane^47^. To confirm that the observed down-regulation of genes involved in mitochondrial respiration was associated with corresponding changes in protein expression, we performed Western blotting on mitochondrial complexes using an antibody cocktail (Total OXPHOS Human WB Antibody Cocktail (ab110411), abcam, UK), recognizing epitopes of purified subunits of II (SDHB), III (UQCRC2), IV (MTCOX1), and V (ATP5α). Total OXPHOS Human WB Antibody Cocktail did not detect Complex I (NDUFB8) in PHT cell lysates so we used Anti-NDUFB8 antibody (ab155903) for this purpose. Inhibition of mTORC1 decreased the expression levels of subunits of complexes I, II, III, IV and V as compared to control (n = 4/each group; Fig. 1). In contrast, protein expression of mitochondrial complexes was unaffected by mTORC2 inhibition (n = 4/each group; Fig. 1).

### Effect of mTORC1 inhibition on TOM20 (translocase of the outer membrane 20) protein expression in PHT cells

Biogenesis of functional and competent mitochondria requires the import and assembly of proteins synthesized in the cytoplasm. Mitochondria contain more than 1000 proteins that are imported from the cytoplasm through the translocase of the outer mitochondrial membrane (TOM complex). Proteins to be translocated into the mitochondria are targeted by the presequence receptors Tom20 or Tom70 and enter the mitochondria via the pore, which is mainly formed by Tom40^48^. Cytochrome b (Cyt b) is an integral part of Complex III, and in its absence the entire complex will fail to assemble resulting in a complete inhibition of oxidative phosphorylation. Cytochrome c is associated with the inner membrane of the mitochondria and interacts with redox partners of complex III and complex IV, which is a part of the mitochondrial electron transfer chain. We determined the expression levels of TOM20, cytochrome b and c, which are central for the regulation of mitochondrial function, following mTOR inhibition in PHT cells. As shown in Supplemental Fig. 4, inhibition of mTORC1 signaling decreased the expression of TOM20, cytochrome b and c as compared to control (n = 6/each group; Supplemental Fig. 4).

### Inhibition of mTORC1 decreases mitochondrial respiration in cultured PHT cells

**Figure 2:**
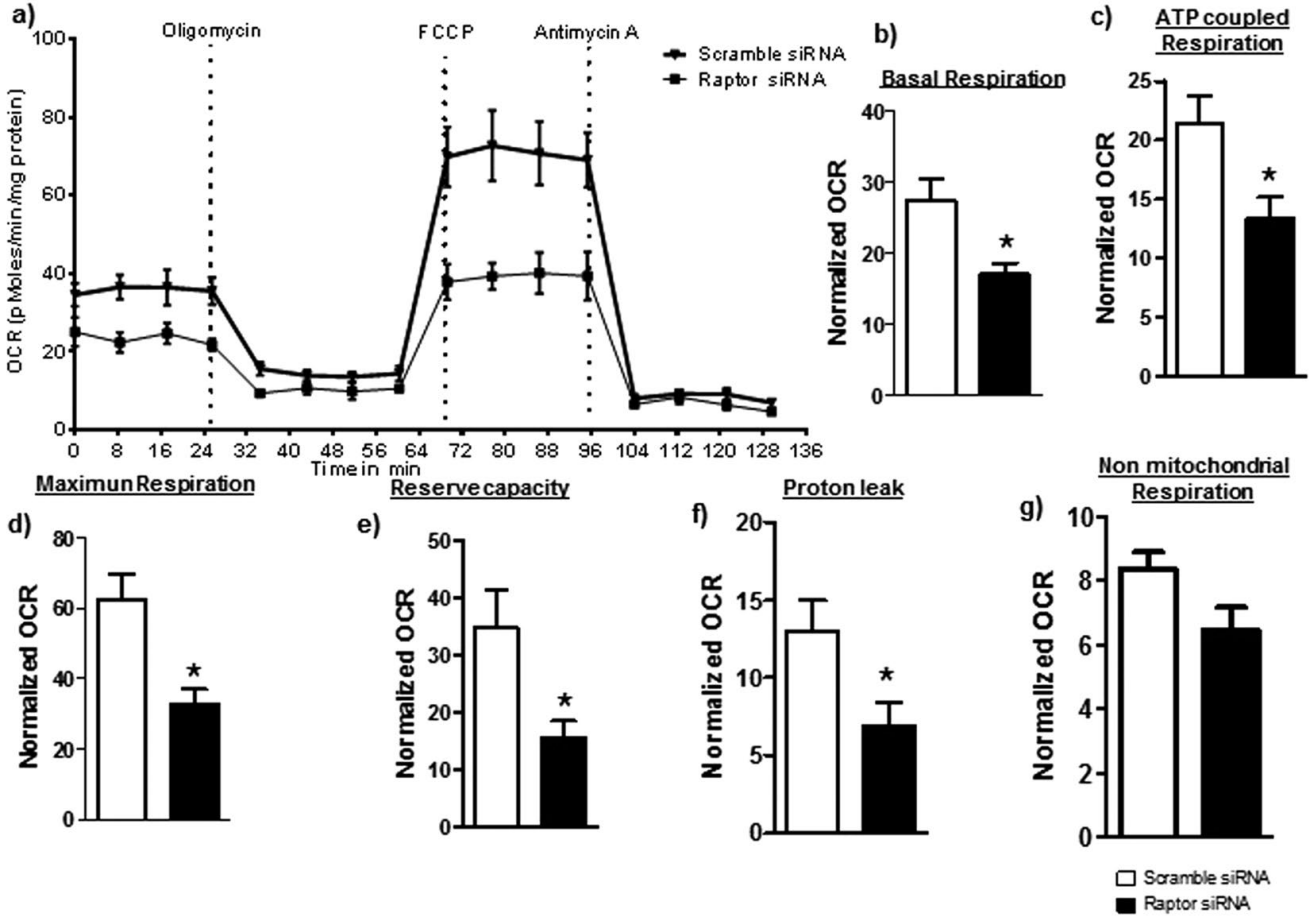
Effect of mTORC1 inhibition on mitochondrial respiration in PHT cells. Mitochondrial respiration was measured in PHT cells at 90 hours of culture following inhibition of mTORC1 by silencing of raptor. Individual oxygen consumption rate (OCR) parameters (basal, ATP-coupled and maximal respiration, non-mitochondrial respiration, reserve capacity and proton leak) were calculated based on total cellular protein and expressed relative to control PHT cells (transfected with scramble siRNA). Data are presented as mean + S.E.M. *P < 0.05 vs scramble siRNA cells, paired student’s T-test, n = 4 independent experiments.

We assessed mitochondrial function in PHT cells by determining OCR following sequential addition of pharmacological inhibitors to probe the function of individual components of the respiratory chain. The design of these experiment is provided in Supplemental Methods. To estimate the proportion of the basal OCR coupled to ATP synthesis, ATP synthase (Complex V) was inhibited by oligomycin, which decreases the OCR rate to the extent to which the cells are using mitochondria to generate ATP. The remaining OCR is ascribed to proton leak across the mitochondrial membrane. To determine the maximal OCR that the cells can sustain, the proton ionophore FCCP was injected. Lastly, antimycin A was injected to inhibit electron flux through Complex III which causes a dramatic suppression of the OCR. The remaining OCR is attributable to O_2_ consumption due to the formation of mitochondrial ROS and non-mitochondrial sources. The reserve respiratory capacity was calculated as the maximal rate minus the basal rate and presents a parameter which is available to cells for increased work to cope with stress. As shown in Fig. 2 (n = 4/each group), inhibition of mTORC1 decreased the basal respiration (p = 0.02), ATP-coupled respiration (p = 0.04), maximum respiration (p = 0.01), reserve capacity (p = 0.04) and proton leak (p = 0.04) as compared to control. Non-mitochondrial respiration was not significantly affected (p = 0.08) by mTORC1 inhibition (Fig. 2).

### No effect of mTORC2 inhibition on mitochondrial respiration in cultured PHT cells

**Figure 3:**
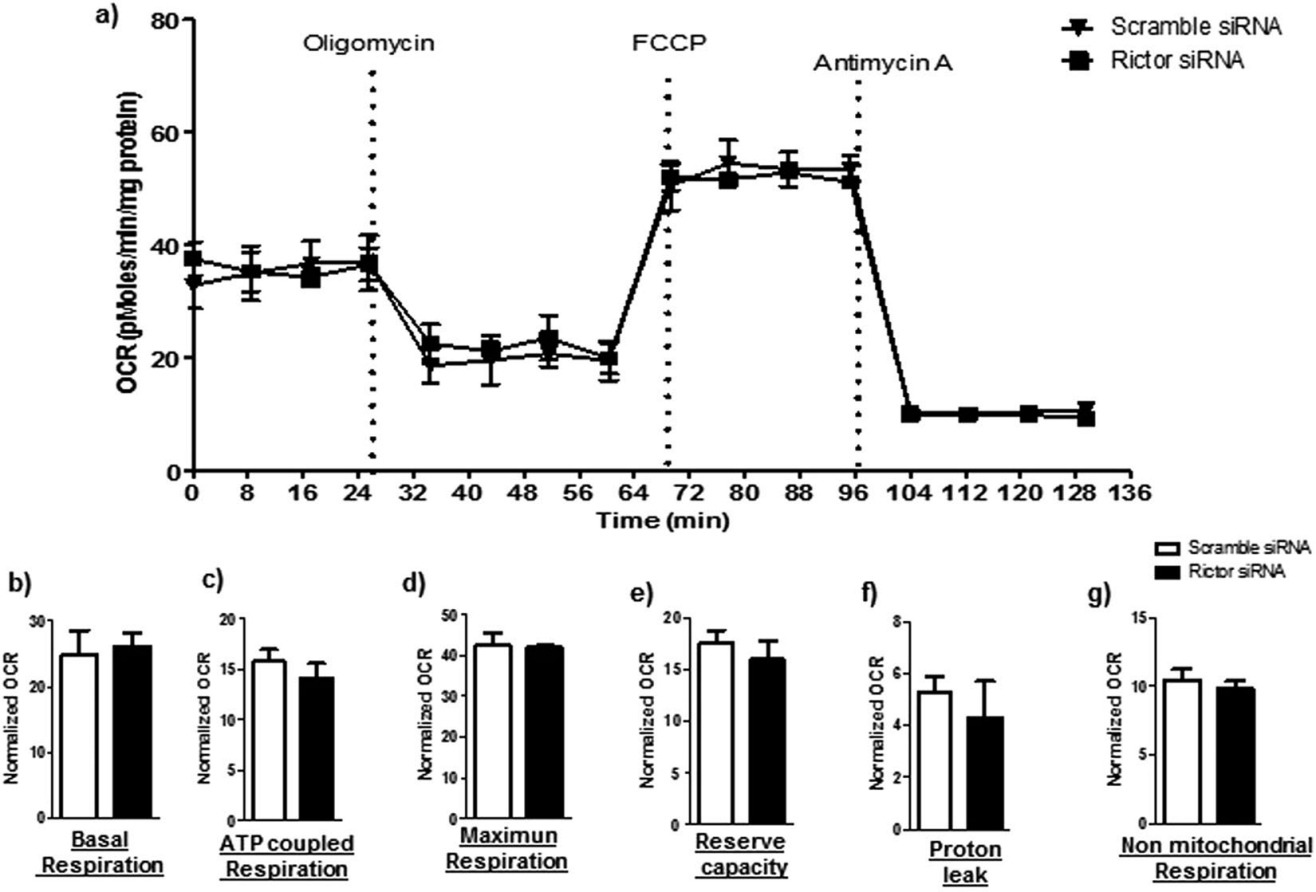
Effect of mTORC2 inhibition on mitochondrial function in PHT cells. Mitochondrial respiration was measured in PHT cells at 90 hours of culture following inhibition of mTORC2 by silencing of rictor. Individual oxygen consumption rate (OCR) parameters (basal, ATP-coupled and maximal respiration, non-mitochondrial respiration, reserve capacity and proton leak) were calculated based on total cellular protein and expressed relative to control PHT cells (transfected with scramble siRNA). Data are presented as mean + S.E.M., paired student’s T-test, n = 4 independent experiments.

We also studied the effects of mTORC2 inhibition on mitochondrial function in cultured PHT cells. Basal, ATP-coupled and maximum respiration, as well as reserve capacity, non-mitochondrial respiration, and proton leak were unaffected by mTORC2 inhibition by rictor silencing (n = 4/each group; Fig. 3).

### Activation of mTORC1 and 2 increases mitochondrial respiration in cultured PHT cells

**Figure 4:**
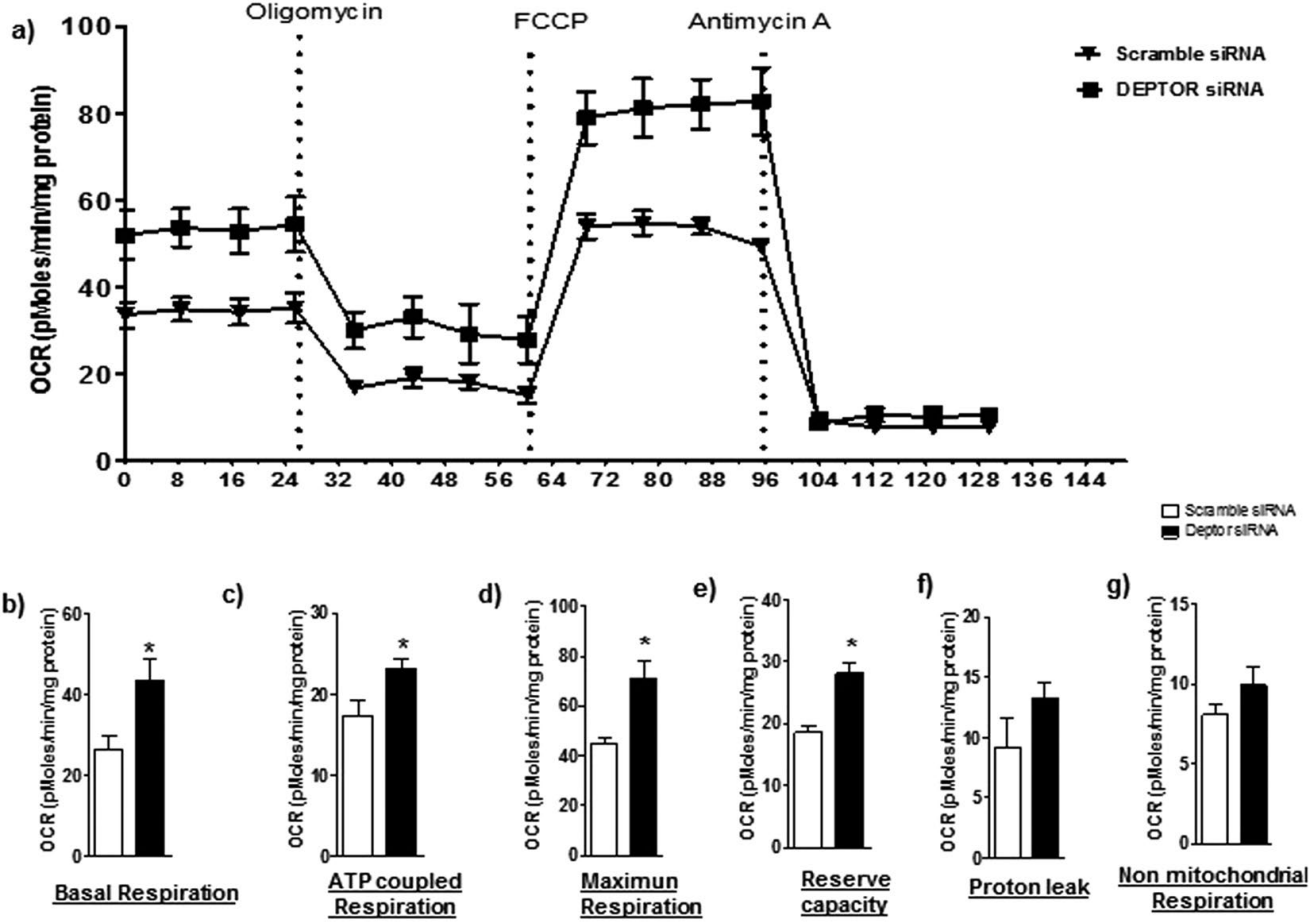
Effect of activation of mTORC1 and 2 on mitochondrial respiration in PHT cells. Mitochondrial respiration was measured in PHT cells at 90 hours of culture following activation of mTORC1/2 by silencing of DEPTOR. Individual oxygen consumption rate (OCR) parameters (basal, ATP-coupled and maximal respiration, non-mitochondrial respiration, reserve capacity and proton leak) were calculated based on total cellular protein and expressed as relative to PHT cells (transfected with scramble siRNA). Data are presented as mean + S.E.M. *P < 0.05 vs scramble siRNA cells, paired student’s T-test, n = 4 independent experiments.

Activation of mTORC1 and mTORC2 using DEPTOR silencing significantly increased basal respiration (p = 0.03), ATP-coupled respiration (p = 0.03), maximum respiration (p = 0.009), and reserve capacity (p = 0.005) as compared to control. Non-mitochondrial respiration (p = 0.2) and proton leak (p = 0.2) were unaffected by activating mTORC1 and mTORC2 (n = 4/each group; Fig. 4).

### Effect of mTOR on cellular ATP levels

**Figure 5:**
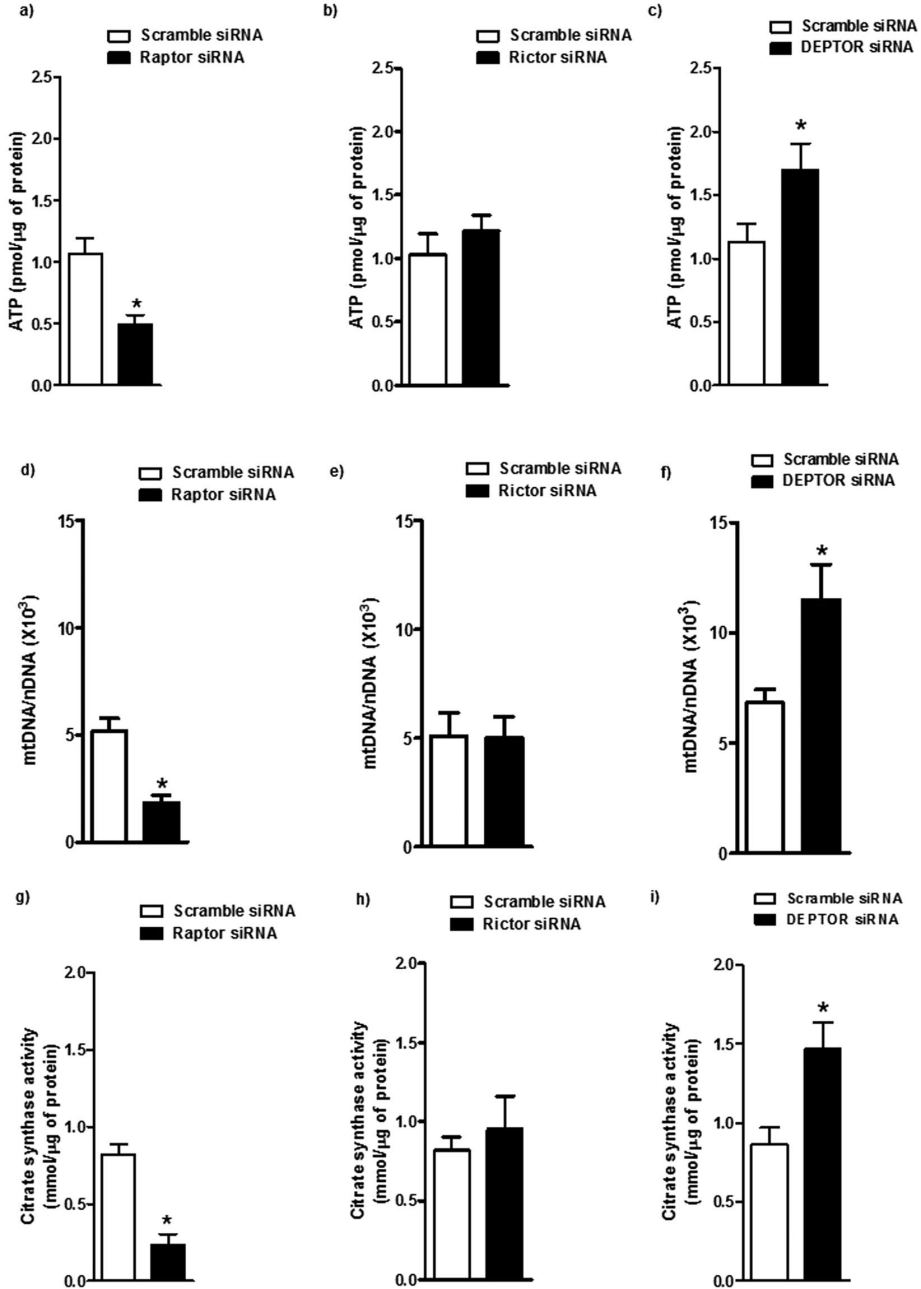
Regulation of PHT cell ATP levels, mitochondrial DNA (mtDNA) copy number, and citrate synthase activity by mTOR signaling. (**a**–**c**) ATP levels were normalized to total protein level of cell lysates. (**d**–**f**) Mitochondrial numbers assessed by mtDNA copy number. mtDNA copy number was normalized to nuclear DNA (nDNA) (β2-microglobulin) for each sample. (**g**–**i**) Mitochondrial numbers estimated by citrate synthase activity. Data are presented as mean + S.E.M. *P < 0.05 vs scramble siRNA cells, n = 6 independent experiments. The number of experiments (**n**) represents the number of placentas studied.

The effect of mTOR inhibition on PHT cell ATP levels was examined. The cellular ATP content was decreased by 50% in raptor silenced cells as compared to control (P < 0.01, n = 6/each group; Fig. 5a). However, the cellular ATP levels were comparable between control and rictor silenced PHT cells (Fig. 5b). In contrast, ATP levels were significantly (P < 0.05, n = 6/each group) increased in mTOR activated PHT cells (DEPTOR silenced) as compared to control (Fig. 5c).

### Mitochondrial DNA copy number

To explore the mechanism that may account for the decline in PHT cellular ATP levels following mTORC1 inhibition, we utilized a RT-PCR approach to measure changes in mitochondrial biogenesis (Fig. 5d–f). The mitochondrial DNA copy number was significantly decreased in raptor silenced cells as compared to control (P < 0001, n = 6/each group; Fig. 5d), whereas mTORC2 inhibition (rictor silencing) did not affect the mitochondrial biogenesis in PHT cells (Fig. 5e). In DEPTOR silenced cells, mitochondrial DNA copy number was significantly increased as compared to control (P < 0.05, n = 6/each group; Fig. 5f).

### Mitochondrial citrate synthase activity

We also estimated the mitochondrial content by measuring citrate synthase activity. mTORC1 inhibition showed a significant reduction in citrate synthase activity (P < 0.0001, n = 6/each group; Fig. 5g), whereas citrate synthase activity was comparable between rictor silenced and control PHT cells (Fig. 5h). DEPTOR silencing significantly increased the citrate synthase activity as compared to control (P < 0.01, n = 6/each group; Fig. 5i).

### Visualization of mitochondria in PHT cells using immunofluorescence

**Figure 6:**
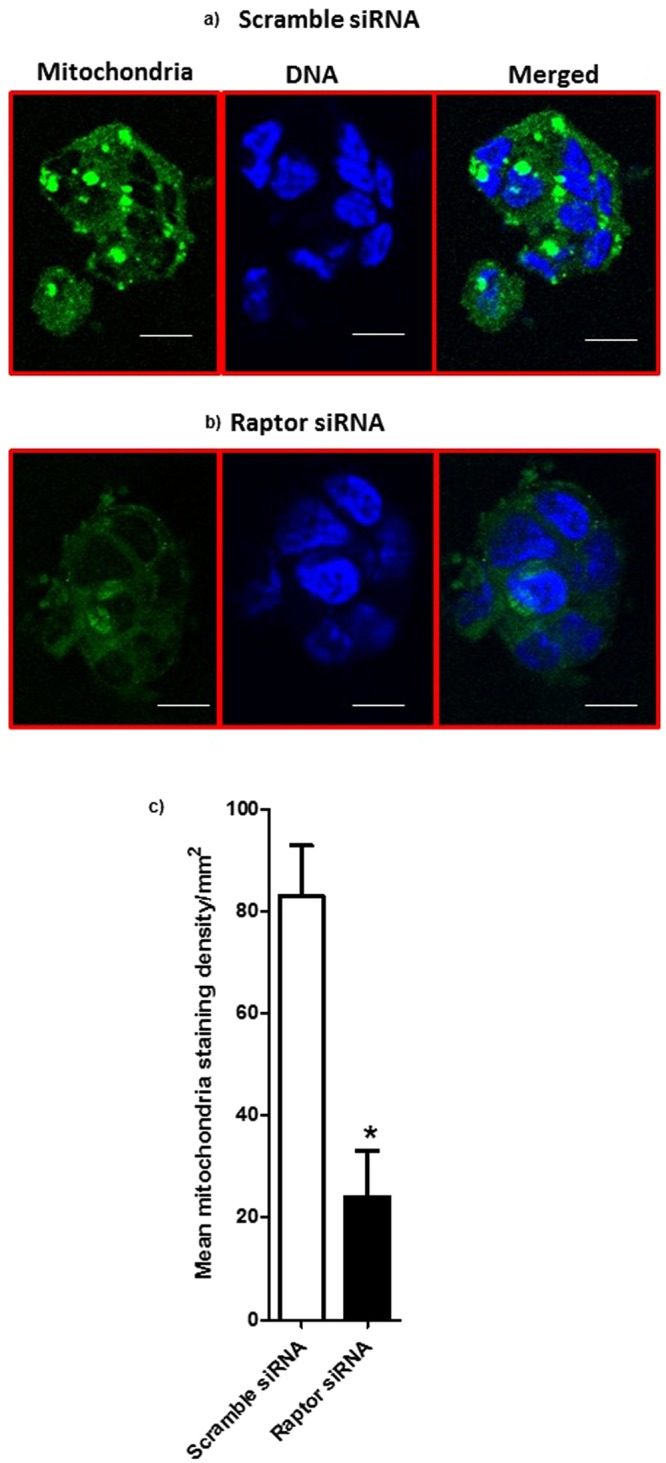
Visualization of mitochondria in PHT cells using immunofluorescence. Trophoblast cells were transfected at 18 h in culture with scramble (**a**) or raptor (**b**) siRNA. At 90 h in culture, cells were incubated with mitochondrial dye and fixed and mitochondria (green) were visualized using immunofluorescence. The data are from a representative experiment, and similar results were obtained from three other experiments. Nuclei were counterstained using DAPI (4′,6-diamidino-2-phenylindole) (blue); scale bar 50 µm. (**c**) Histogram summarizes the syncytiotrophoblast mitochondrial staining intensity in PHT cells transfected with either scramble or raptor siRNA. In each section, at least five randomly selected microscopic fields were used to calculate staining density per mm^2^ using Image J and data were averaged to represent a single placenta. Values are given as mean + SEM; *P < 0.05 vs scramble siRNA; unpaired Student’s t-test; n = 4 placenta/each group.

To provide additional support for the idea that mTORC1 signaling influences mitochondrial biogenesis, we used confocal microscopy to visualize mitochondria in cultured PHT cells. We observed that in response to raptor silencing mitochondria content decreased as compared to control PHT cells (Fig. 6).

### Placental mTORC1 signaling is associated with the protein expression of mitochondrial ETC complexes in human pregnancy

**Figure 7:**
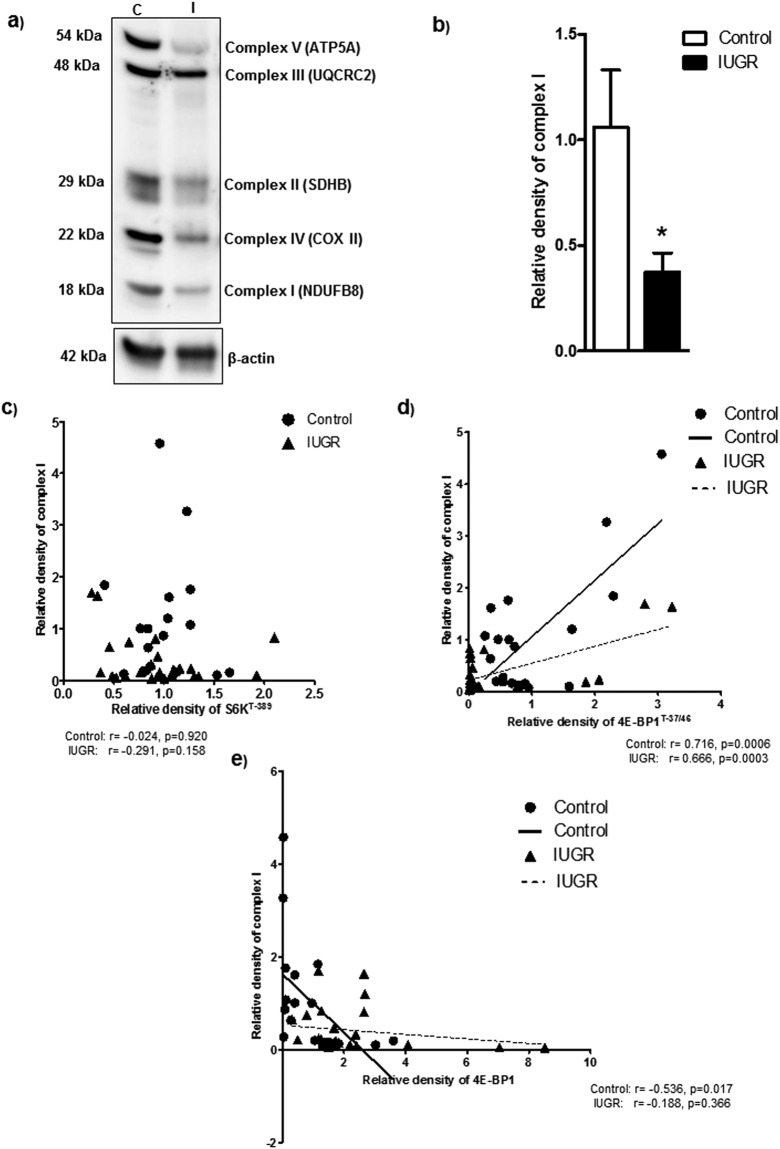
Correlation between placental mTORC1 functional readouts and protein expression of mitochondrial ETC complex I. (**a**) Protein expression of mitochondrial electron transport chain complexes in placental homogenates of control and IUGR group. Representative Full length Western blot is shown. (**b**) Relative expression of mitochondrial electron transport chain complex I expression in placental homogenates of control and IUGR. After normalization to β-actin, the mean density of C samples was assigned an arbitrary value of 1. Subsequently, individual IUGR density values were expressed relative to this mean. (**c**–**e**) Correlation between placental mTORC1 functional readouts S6K^T-389^ (**c**), 4E-BP1 ^T-37/46^ (**d**) 4E-BP1 (**e**) and mitochondrial ETC complex I expression. r = Pearson correlation coefficient, n = Control, 19; IUGR, 25. Values are given as means ± S.E.M.; *P < 0.05 vs. control; unpaired Student’s t test.

To explore the clinical relevance of our findings, we examined the relationship between placental mTORC1 signaling and the protein expression of mitochondrial electron transport chain complexes in placentas collected from control and IUGR pregnancies. The protein expression of ETC complex I (Fig. 7a) and II-V (Supplementary Figs 5a–8a) were significantly reduced in IUGR placentas. Placental raptor and rictor expressions were significantly decreased in the IUGR as compared to control (Supplementary Fig. 9). In contrast, placental DEPTOR expression was increased in the IUGR group as compared to control (Supplementary Fig. 9). Placental mTORC1 functional readouts signaling in the same placentas has been reported elsewhere^20^. With few exceptions, placental mTORC1 signaling functional readouts of phosphorylated 4E-BP1 (Thr-37/46) and S6 kinase (Thr-389) were positively correlated with mitochondrial ETC complexes (Fig. 7 and Supplementary Figs 5–8). The 4E-BP1 protein prevents translation initiation by binding eukaryotic translation initiation factor 4E (eIF4E) and phosphorylation of 4E-BP1 interferes with this binding, thereby activating protein translation. As a consequence, both the increased total expression of 4E-BP1 and the decreased phosphorylation of 4E-BP1 at Thr37/46 will promote binding of eIF4E resulting in inhibition of cap-dependent translation initiation in the IUGR placenta. Placental 4E-BP1 expression negatively correlated with mitochondrial ECT complexes I and V (Fig. 7 and Supplementary Figs 5–8).

## Discussion

We report that mTORC1, but not mTORC2, is a positive regulator of mitochondrial oxidative phosphorylation in primary human trophoblast cells mediated by influencing mitochondrial biogenesis. These findings extend previous reports of mTORC1 regulation of mitochondrial respiration in cell lines to human primary non-proliferative cells. In addition, we provide evidence that placental protein expression of ETC complexes was decreased and positively correlated to mTOR signaling activity in IUGR. By controlling trophoblast ATP production, which is essential for energizing active transport and protein synthesis, mTORC1 links nutrient and O_2_ availability and growth factor signaling to placental function and fetal growth. Reduced placental mTOR activity may impair mitochondrial respiration and contribute to placental insufficiency in IUGR pregnancies.

The use of PHT cells contributes to the relevance of our studies to human physiology and the demonstration of decreased protein expression of mitochondrial ETC complexes in human IUGR placentas highlights the potential clinical impact of our findings. Although mTOR is known as the master regulator of protein translation, our data show that mTOR has a profound influence on trophoblast mitochondrial ETC gene expression, with mTORC1 and mTORC2 showing differential regulation. Importantly, mTORC1, but not mTORC2, constitutes a positive regulator of trophoblast mitochondrial respiration mediated by changes in the expression of genes representing all five complexes of the electron transport chain. By controlling trophoblast production of ATP, which is critical for active transport and protein synthesis, and regulating plasma membrane trafficking of amino acid transporters^25^, mTORC1 links nutrient and oxygen availability and growth factor signaling to placental function. Our findings are consistent with the possibility that reduced placental mTOR activity in IUGR^18–20^ may impair mitochondrial respiration and contribute to placental insufficiency in this pregnancy complication. However, the available literature assessing mitochondrial DNA and expression of ETC complexes in the human IUGR placenta is not entirely consistent. For example, whereas some investigators have reported that IUGR is associated with decreased placental mitochondrial DNA content^49,50^, others found higher mitochondrial DNA content in the human IUGR placenta^51–53^. Furthermore, Mando^52^ and coworkers reported that mtDNA content and expression of genes encoding for ETC proteins were decreased whereas ETC protein expression was unaltered and ETC activity was increased in cultured trophoblast cells isolated from IUGR placentas^52^. The reasons for the divergent findings in the different studies remains to be established.

Inhibition of mTORC1 resulted in a highly coordinated transcriptional response of genes related to oxidative phosphorylation, which is consistent with the finding that mitochondrial function was the top-ranked pathway affected by raptor silencing. Specifically, genes in all mitochondrial complexes were down-regulated in PHT cells following mTORC1 inhibition. This effect is likely to be mediated, at least in part, by mTORC1 inhibition of mitochondrial biogenesis, as evidenced by decreased cellular ATP levels, citrate synthase activity and mitochondrial copy number in raptor silenced cells.

Previous studies show that muscle-specific knockout of raptor in mice results in down-regulation of mitochondrial gene expression and decreased oxidative capacity^29^. Furthermore, rapamycin treatment or knockdown of mTOR or raptor in muscle cells or in MCF7 cells decreases mitochondrial gene expression and oxygen consumption^27,45,46^. Tuberous sclerosis complex (TSC) 1 and 2 form a heterodimeric complex and inactivate Ras homolog enriched in brain, resulting in inhibition of mTORC1. TSC2^−/−^ knock out in MEFs exhibited hyperactivation of mTORC1 signaling^54^ and an increased expression of mitochondrial genes that were downregulated by rapamycin^27^. Loss of TSC2 in MEF’s increased mitochondrial respiration and mitochondrial DNA content and ATP levels^46^. Furthermore, in the absence of DEPTOR, mTORC1 remains active, which promotes liver mitochondrial oxidative metabolism. Consistent with previous findings^27,46^, in the present study, we found that mTOR activation by silencing DEPTOR in PHT cells increased the mitochondrial oxidative phosphorylation and ATP synthesis. These results further support the finding that mTORC1 signaling positively controls mitochondrial gene expression and oxygen consumption in an mTORC2 independent manner. Taken together, our data in primary human trophoblast cells and previous studies in other cell systems demonstrate that mTORC1 controls mitochondrial respiration, thereby linking increased nutrient availability and activation of growth factor signaling to increased capacity for cellular energy production in an integrated response to promote cellular growth.

Mitochondrial biogenesis also involves an increase of mitochondrial import^55^. In differentiating C2C12 cells overexpression of Tom20 increases and the inhibition of Tom20 decreases the mitochondrial import, demonstrating the critical importance of Tom20 in determining the mitochondrial capacity to important cytosolic proteins^56^. Furthermore, the use of anti-Tom20 antibodies reduces the import of several mitochondrial proteins^57^. In our study, the mitochondrial Tom20 protein level was reduced by mTORC1 inhibition, which may be secondary to the decreased mitochondrial number and/or as a result of mTORC1 directly regulating Tom20 expression.

To our knowledge, this is the first study showing mTORC1 regulation of mitochondrial function in trophoblast cells. However, the molecular mechanisms linking mTORC1 inhibition to decreased mitochondrial biogenesis in PHT cells remain to be established. Long-term inhibition of mTORC1 with rapamycin was found to decrease PGC-1α (Peroxisome-proliferator-activated receptor coactivator-1α) mediated gene transcription in muscle cells *in vitro*, possibly mediated by the yin-yang 1 (YY1) transcription factor^27^. As a result, mitochondrial DNA content and oxygen consumption were lowered^27^. Reduction in energetic capacity renders cells more susceptible to stressors^58^.

Placental mitochondrial dysfunction has been proposed to represent one mechanism underlying fetal programming in cases of placental insufficiency^5^. In mouse embryos, altered mitochondrial function affects fetal and placental growth^59^, and conversely, restricted fetal growth induced by maternal under-nutrition in rats is associated with impaired placental mitochondrial function^6^. A multitude of nutrient transporters, including amino acid, vitamin and calcium transporters, mediate active transport across the placental barrier and therefore requires a continuous supply of ATP. Although a significant portion of trophoblast ATP is produced by the glycolytic pathway, the majority is likely to be derived from mitochondrial respiration. Thus, impaired mitochondrial function and decreased placental ATP production^3^, may link placental mTORC1 inhibition in placental insufficiency^18,22^ to restricted fetal growth.

We demonstrated that protein expression of mitochondrial ETC complexes were decreased in human IUGR placentas. In addition, placental mTORC1 signaling functional readouts were positively correlated with the protein expression of placental mitochondrial ETC complexes. These findings suggest that our study has relevance to clinically important pregnancy complications. In addition, given that trophoblast mitochondrial respiration can be directly modulated by mTOR signaling, we speculate that activation of placental mTOR signaling to promote energy production may be a target of interest in future development of novel intervention strategies in IUGR.

**Figure 8:**
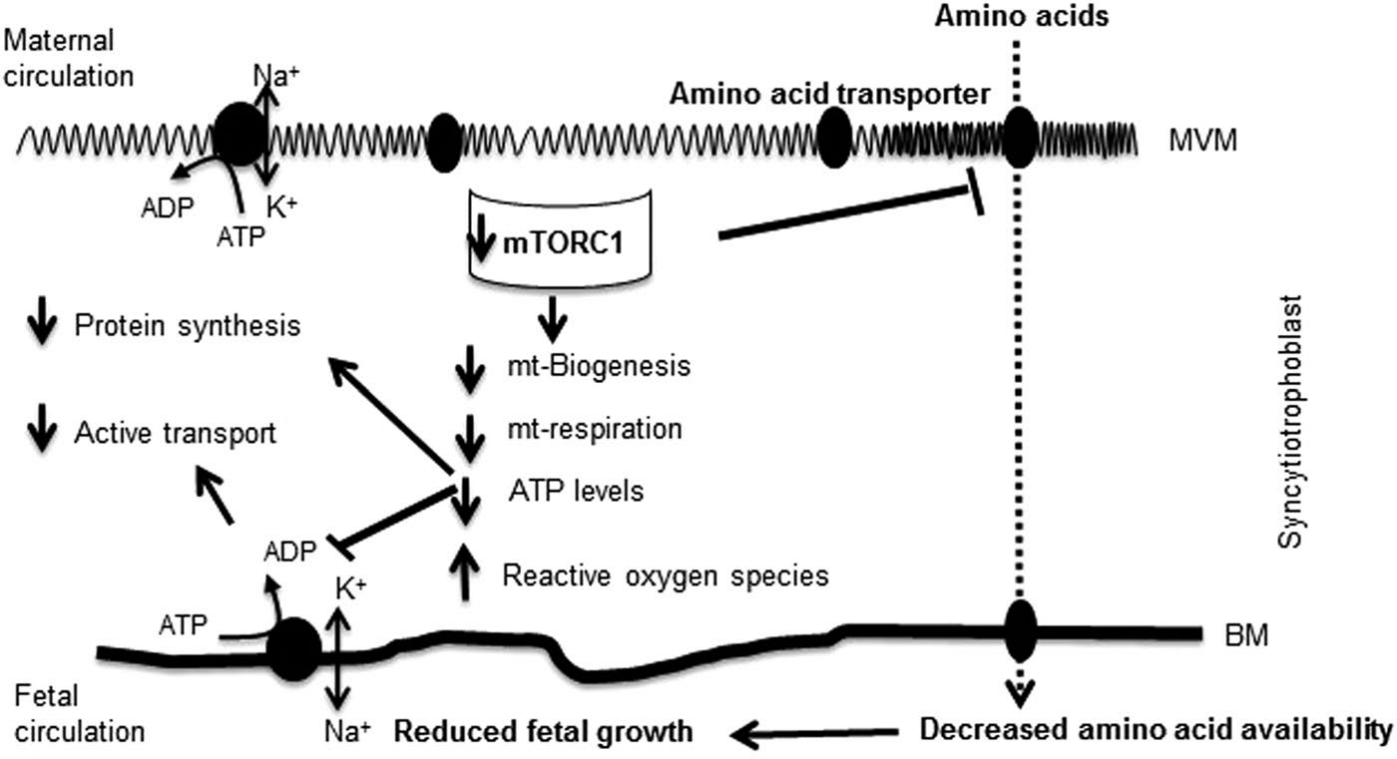
A proposed model linking mTORC1 inhibition, mitochondrial respiration and fetal growth restriction. Placental mTORC1 signaling is inhibited in fetal growth restriction, we speculate that decreased trophoblast mitochondrial respiration and ATP production may link placental mTORC1 inhibition in placental insufficiency to restricted fetal growth. Abbreviation: MVM: Microvillus plasma membrane; BM: Basal plasma membrane; mt: Mitochondria.

In conclusion, mTORC1, but not mTORC2, is a positive regulator of the trophoblast expression of genes encoding ETC proteins and mitochondrial respiratory function, likely mediated by effects on mitochondrial biogenesis. By controlling trophoblast production of ATP, which is critical for active transport and protein synthesis, and regulating plasma membrane trafficking of amino acid transporters^25^, mTOR functions as a placental nutrient sensor linking nutrient and oxygen availability and growth factor signaling to placental function and fetal growth. Because placental mTORC1 signaling is inhibited in fetal growth restriction, we speculate that decreased mitochondrial respiration induced by inhibition of mTORC1 signaling directly contributes to placental insufficiency in this pregnancy complication (Fig. 8).

## Electronic supplementary material

Supplemental Materials

## References

[1] Martinez F, Olvera-Sanchez S, Esparza-Perusquia M, Gomez-Chang E, Flores-Herrera O (2015). Multiple functions of syncytiotrophoblast mitochondria. Steroids.

[2] von Kleist-Retzow JC (2003). Antenatal manifestations of mitochondrial respiratory chain deficiency. J Pediatr.

[3] Owens JA, Falconer J, Robinson JS (1987). Effect of restriction of placental growth on fetal and utero-placental metabolism. J Dev Physiol.

[4] Chiaratti MR (2015). Is Placental Mitochondrial Function a Regulator that Matches Fetal and Placental Growth to Maternal Nutrient Intake in the Mouse?. PLoS One.

[5] Leduc L, Levy E, Bouity-Voubou M, Delvin E (2010). Fetal programming of atherosclerosis: possible role of the mitochondria. Eur J Obstet Gynecol Reprod Biol.

[6] Mayeur S (2013). Maternal calorie restriction modulates placental mitochondrial biogenesis and bioenergetic efficiency: putative involvement in fetoplacental growth defects in rats. Am J Physiol Endocrinol Metab.

[7] Poston L (2011). Role of oxidative stress and antioxidant supplementation in pregnancy disorders. Am J Clin Nutr.

[8] Mele J, Muralimanoharan S, Maloyan A, Myatt L (2014). Impaired mitochondrial function in human placenta with increased maternal adiposity. Am J Physiol Endocrinol Metab.

[9] Peng T, Golub TR, Sabatini DM (2002). The Immunosuppressant Rapamycin Mimics a Starvation-Like Signal Distinct from Amino Acid and Glucose Deprivation. Mol. Cell. Biol..

[10] Tee AR, Blenis J (2005). mTor, translational control and human disease. Sem Cell Dev Biol.

[11] Martin DE, Hall MN (2005). The expanding TOR network. Curr Opin Cell Biol.

[12] Hay N, Soneneberg N (2004). Upstream and downstream of mTOR. Genes Dev.

[13] Jacinto E, Hall MN (2003). TOR signalling in bugs, brain and brawn. Nature Rev Mol Cell Biol.

[14] Laplante M, Sabatini DM (2012). mTOR signaling in growth control and disease. Cell.

[15] Alessi DR, Pearce LR, Garcia-Matinez JM (2009). New insights into mTOR signaling: mTORC2 and beyond. Sci Signaling.

[16] Jacinto E (2004). Mammalian TOR complex 2 controls the actin cytoskeleton and is rapamycin insensitive. Nat Cell Biol.

[17] Peterson TR (2009). DEPTOR is an mTOR inhibitor frquently overexpressed in multiple myeloma cells and required for their survival. Cell.

[18] Roos S (2007). Mammalian target of rapamycin in the human placenta regulates leucine transport and is down-regulated in restricted fetal growth. J Physiol.

[19] Yung, H. W. *et al*. Evidence of placental translation inhibition and endoplasmic reticulum stress in the etiology of human intrauterine growth restriction. *Am J Pathol* **173**, 451-462 (2008). 10.2353/ajpath.2008.071193 PMC2475782 18583310

[20] Chen, Y. Y. *et al*. Increased ubiquitination and reduced plasma membrane trafficking of placental amino acid transporter SNAT-2 in human IUGR. *Clin Sci* (*Lond*) (2015). 10.1042/CS20150511 PMC4614027 26374858

[21] Rosario FJ (2011). Maternal protein restriction in the rat inhibits placental insulin, mTOR, and STAT3 signaling and down-regulates placental amino acid transporters. Endocrinology.

[22] Kavitha JV (2014). Down-regulation of placental mTOR, insulin/IGF-I signaling, and nutrient transporters in response to maternal nutrient restriction in the baboon. FASEB J.

[23] Jansson N (2013). Activation of Placental mTOR Signaling and Amino Acid Transporters in Obese Women Giving Birth to Large Babies. J Clin Endocrinol Metab.

[24] Rosario FJ, Kanai Y, Powell TL, Jansson T (2015). Increased placental nutrient transport in a novel mouse model of maternal. obesity with fetal overgrowth. Obesity (Silver Spring).

[25] Rosario FJ, Kanai Y, Powell TL, Jansson T (2013). Mammalian target of rapamycin signalling modulates amino acid uptake by regulating transporter cell surface abundance in primary human trophoblast cells. J Physiol.

[26] Rosario FJ, Powell TL, Jansson T (2016). Mechanistic target of rapamycin (mTOR) regulates trophoblast folate uptake by modulating the cell surface expression of FR-alpha and the RFC. Sci Rep.

[27] Cunningham JT (2007). mTOR controls mitochondrial oxidative function through a YY1-PGC-1alpha transcriptional complex. Nature.

[28] Koyanagi M (2011). Ablation of TSC2 enhances insulin secretion by increasing the number of mitochondria through activation of mTORC1. PloS one.

[29] Bentzinger, C. F. *et al*. Skeletal muscle-specific ablation of raptor, but not of rictor, causes metabolic changes and results in muscle dystrophy. *Cell Metab* **411**–**424** (2008). 10.1016/j.cmet.2008.10.002 19046572

[30] Shende P (2011). Cardiac raptor ablation impairs adaptive hypertrophy, alters metabolic gene expression, and causes heart failure in mice. Circulation.

[31] Ramanathan A, Schreiber SL (2009). Direct control of mitochondrial function by mTOR. Proc Natl Acad Sci USA.

[32] Kliman HJ, Nestler JE, Sermasi E, Sanger JM, Strauss JF (1986). Purification, characterization, and *in vitro* differentiation of cytotrophoblasts from human term placentae. Endocrinology.

[33] Li L, Schust DJ (2015). Isolation, purification and *in vitro* differentiation of cytotrophoblast cells from human term placenta. Reprod Biol Endocrinol.

[34] Petroff MG, Phillips TA, Ka H, Pace JL, Hunt JS (2006). Isolation and culture of term human trophoblast cells. Methods Mol Med.

[35] Chen YY, Powell TL, Jansson T (2017). 1,25-Dihydroxy vitamin D3 stimulates system A amino acid transport in primary human trophoblast cells. Mol Cell Endocrinol.

[36] Rosario FJ, Powell TL, Jansson T (2017). mTOR folate sensing links folate availability to trophoblast cell function. J Physiol.

[37] Forbes K, Desforges M, Garside R, Aplin JD, Westwood M (2009). Methods for siRNA-mediated reduction of mRNA and protein expression in human placental explants, isolated primary cells and cell lines. Placenta.

[38] Kanehisa M, Furumichi M, Tanabe M, Sato Y, Morishima K (2017). KEGG: new perspectives on genomes, pathways, diseases and drugs. Nucleic Acids Res.

[39] Kanehisa M, Sato Y, Kawashima M, Furumichi M, Tanabe M (2016). KEGG as a reference resource for gene and protein annotation. Nucleic Acids Res.

[40] Kanehisa M, Goto S (2000). KEGG: kyoto encyclopedia of genes and genomes. Nucleic Acids Res.

[41] Bayerlova M (2015). Comparative study on gene set and pathway topology-based enrichment methods. BMC Bioinformatics.

[42] Doniger SW (2003). MAPPFinder: using Gene Ontology and GenMAPP to create a global gene-expression profile from microarray data. Genome Biol.

[43] Ogata H (1999). KEGG: Kyoto Encyclopedia of Genes and Genomes. Nucleic Acids Res.

[44] Maloyan A, Mele J, Muralimanohara B, Myatt L (2012). Measurement of mitochondrial respiration in trophoblast culture. Placenta.

[45] Schieke SM (2006). The mammalian target of rapamycin (mTOR) pathway regulates mitochondrial oxygen consumption and oxidative capacity. J Biol Chem.

[46] Morita M (2013). mTORC1 controls mitochondrial activity and biogenesis through 4E-BP-dependent translational regulation. Cell Metab.

[47] Duchen MR, Szabadkai G (2010). Roles of mitochondria in human disease. Essays Biochem.

[48] Yano M, Terada K, Mori M (2004). Mitochondrial import receptors Tom20 and Tom22 have chaperone-like activity. J Biol Chem.

[49] Diaz M (2014). Mitochondrial DNA in placenta: associations with fetal growth and superoxide dismutase activity. Horm Res Paediatr.

[50] Poidatz D (2015). Involvement of estrogen-related receptor-gamma and mitochondrial content in intrauterine growth restriction and preeclampsia. Fertil Steril.

[51] Lattuada D (2008). Higher mitochondrial DNA content in human IUGR placenta. Placenta.

[52] Mando C (2014). Placental mitochondrial content and function in intrauterine growth restriction and preeclampsia. Am J Physiol Endocrinol Metab.

[53] Novielli C (2017). Mitochondrial DNA content and methylation in fetal cord blood of pregnancies with placental insufficiency. Placenta.

[54] Zhang H (2003). Loss of Tsc1/Tsc2 activates mTOR and disrupts PI3K-Akt signaling through downregulation of PDGFR. J Clin Invest.

[55] Takahashi M, Chesley A, Freyssenet D, Hood DA (1998). Contractile activity-induced adaptations in the mitochondrial protein import system. Am J Physiol.

[56] Grey JY (2000). Tom20-mediated mitochondrial protein import in muscle cells during differentiation. Am J Physiol Cell Physiol.

[57] Millar DG, Shore GC (1996). Signal anchor sequence insertion into the outer mitochondrial membrane. Comparison with porin and the matrix protein targeting pathway. J Biol Chem.

[58] Brand MD, Nicholls DG (2011). Assessing mitochondrial dysfunction in cells. Biochem J.

[59] Wakefield SL, Lane M, Mitchell M (2011). Impaired mitochondrial function in the preimplantation embryo perturbs fetal and placental development in the mouse. Biol Reprod.

