# Supplementary material for: Mechanistic Target of Rapamycin Complex 1 Promotes the Expression of Genes Encoding Electron Transport Chain Proteins and Stimulates Oxidative Phosphorylation in Primary Human Trophoblast Cells by Regulating Mitochondrial Biogenesis
Source: Sci Rep. 2019 Jan 22;9:246. doi: 10.1038/s41598-018-36265-8 (PMC6343003; doi:10.1038/s41598-018-36265-8)
Supplement: Supplementary file 1 — Supplemental Materials [file 41598_2018_36265_MOESM1_ESM.pdf]

Mechanistic Target of Rapamycin Complex 1 Promotes the Expression of Genes Encoding Electron Transport Chain Proteins and Stimulates Oxidative Phosphorylation in Primary Human Trophoblast Cells by Regulating Mitochondrial Biogenesis

[1]Fredrick J Rosario, [2]Madhulika B Gupta, [3]Leslie Myatt, [1,4]Theresa L Powell, [5]Jeremy P. Glenn, [5,6] Laura Cox, [1]Thomas Jansson

[1]Division of Reproductive Sciences, Department of OB/GYN, University of Colorado Anschutz Medical Campus, Aurora, CO, USA; [2]Children's Health Research Institute and Department of Pediatrics and Biochemistry, University of Western Ontario, London, Ontario N6A 5C1, Canada; [3]Department of Obstetrics and Gynecology, Oregon Health and Science University, Portland, OR; [4]Section of Neonatology, Department of Pediatrics, University of Colorado Anschutz Medical Campus, Aurora, CO, USA; [5]Department of Genetics, Southwest National Primate Research Center, Texas Biomedical Research Institute, San Antonio, TX and [6]Department of Internal Medicine, Section of Molecular Medicine and Center for Precision Medicine, Wake Forest School of Medicine, Winston-Salem, NC.

Short Title: mTOR and trophoblast mitochondrial function

Key words: Placenta, maternal-fetal exchange, human, energy metabolism, oxidative phosphorylation

Corresponding author:

Fredrick J Rosario

Department of Obstetrics & Gynecology, 12700 East 19th Avenue, University of Colorado Anschutz Medical Campus, Aurora, Colorado 80045.

Office: 303 724 8857, Fax: 303 724 3512.

Email: FREDRICK.JOSEPH@UCDENVER.EDU

Measurement of Oxygen Consumption Rates (OCR) in PHT cells

In brief, PHT cells were metabolically perturbed by additions of three different compounds (in succession) that shift the bioenergetics profile of the cell.

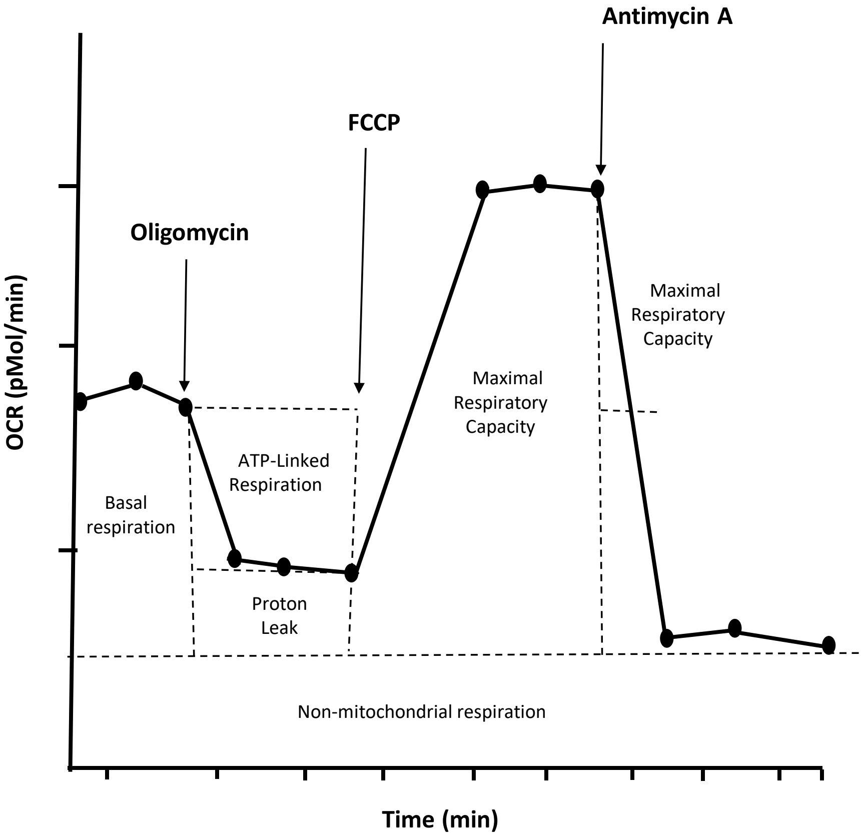

Figure. The Seahorse assay. Oxygen consumption rate (OCR) is measured before and after the addition of inhibitors to derive several parameters of mitochondrial respiration. Initially, baseline cellular OCR is measured, from which basal respiration can be derived by subtracting non-mitochondrial respiration. Next oligomycin, a complex V inhibitor, is added and the resulting OCR is used to derive ATP-linked respiration (by subtracting the oligomycin rate from baseline cellular OCR) and proton leak respiration (by subtracting non-mitochondrial respiration from the oligomycin rate). Next carbonyl cyanide-p-trifluoromethox- yphenyl-hydrazon (FCCP), a protonophore, is added to collapse the inner membrane gradient, allowing the ETC to function at its maximal rate, and maximal respiratory capacity is derived by subtracting non- mitochondrial respiration from the FCCP rate. Lastly, antimycin A and rotenone, inhibitors of complex III and I, are added to shut down ETC function, revealing the non-mitochondrial respiration.

As shown in Figure, initially oligomycin was added. Oligomycin inhibits ATP synthesis by blocking the proton channel of the Fo portion of ATP synthase (Complex V). In mitochondrial research, it is used to prevent state 3 (phosphorylating) respiration. With cells, it can be used to distinguish the percentage of O_2 consumption devoted to ATP synthesis and the percentage of O_2 consumption needed to overcome the natural proton leak across the inner mitochondrial membrane. FCCP (Carbonyl cyanide-p-trifluoromethoxyphenylhydrazone) was the second compound added. FCCP is an ionophore that function as a mobile ion carrier. It is an uncoupling agent because it disrupts ATP synthesis by transporting hydrogen ions across the mitochondrial membrane instead of the proton channel of ATP synthase (Complex V). This collapse of the mitochondrial membrane potential leads to a rapid consumption of energy and oxygen without the generation of ATP resulting in an increase in OCR. FCCP treatment can be used to calculate the "reserve" respiratory capacity of cells that is defined as the difference between maximal uncontrolled OCR and the initial basal OCR. It has been proposed that the maintenance of some reserve respiratory capacity even under conditions of maximal physiological or pathophysiological stimulus is a major factor defining the vitality and/or survival of cells. The ability of cells to respond to stress under conditions of increased energy demand is in large part influenced by the bioenergetic capacity of mitochondria. This bioenergetic capacity is determined by several factors, including the ability of the cell to deliver substrate to mitochondria and the functional capacity of enzymes involved in electron transport. Rotenone, a Complex I inhibitor, was the third compound added,, allowing both the mitochondrial and non-mitochondrial fractions contributing to respiration to be calculated. Rotenone prevents the transfer of electrons from the Fe-S center in Complex I to ubiquinone (Coenzyme Q). This inhibition of Complex I prevents the potential energy in NADH from being converted to usable energy in the form of ATP.

Supplemental Figure 1. Study design.

Supplemental Figure 2. Effect of (a) raptor and (b) rictor silencing on raptor or rictor protein expression respectively in PHT cells. (a) Protein expression of raptor in PHT cells transfected with either scramble or raptor (mTORC1 inhibition) siRNA (n=4/each group). (b) Protein expression of rictor in PHT cells transfected with either scramble or rictor (mTORC2 inhibition) siRNA (n=4/each group). Representative Western blot is shown. The data are from a representative experiment, and similar results were obtained from three other experiments.

Supplemental Figure 3. KEGG pathway analysis for the expression of genes involved in oxidative phosphorylation in PHT cells with mTORC1 inhibition as compared to control PHT cells (transfected with scramble siRNA). Genes are denoted by gene IDs, red indicates down-regulation of gene expression in cells with mTORC1 inhibition compared with control. The KEGG pathway analysis were generated through the use of KEGG (Kyoto Encyclopedia of Genes and Genomes) data base[1].

Cytochrome c oxidase subunit VIIc	COX7C
Ubiquinol-cytochrome c reductase binding protein	UQCRB
Ubiquinol-cytochrome c reductase, complex III subunit VII, 9.5kDa	UQCRQ
Cytochrome c oxidase subunit Vic	COX6C
NADH dehydrogenase (ubiquinone) 1 alpha subcomplex, 1, 7.5kDa	NDUFA1
ATP synthase, H+ transporting, mitochondrial F0 complex, subunit F2	ATP5J2

NADH dehydrogenase (ubiquinone) 1 beta subcomplex, 8, 19kDa	NDUFB8
NADH dehydrogenase (ubiquinone) 1 beta subcomplex, 6, 17kDa	NDUFB6
NADH dehydrogenase (ubiquinone) 1 beta subcomplex, 6, 17kDa	NDUFB6
Succinate dehydrogenase complex, subunit C, integral membrane protein, 15kDa	SDHC
ATPase, H+ transporting V0 subunit e2	ATP6V0E2
NADH dehydrogenase (ubiquinone) Fe-S protein 7, 20kDa (NADH-coenzyme Q reductase)	NDUFS7
ATP synthase, H+ transporting, mitochondrial F0 complex, subunit C1 (subunit 9)	ATP5G1
COX17 cytochrome c oxidase assembly homolog (S. cerevisiae)	COX17

Supplemental Figure 4. Effect of raptor silencing on TOM20, cytochrome b and c protein expression in PHT cells. (a) Protein expression of TOM20, cytochrome b and c in PHT cells transfected with either scramble or raptor (mTORC1 inhibition) siRNA (n=6/each group). Representative Western blot is shown. The data are from a representative experiment, and similar results were obtained from five other experiments.

Supplemental Figure 5. Correlation between placental mTORC1 functional readouts and the protein expression of mitochondrial ETC complex II. (a) Relative expression of mitochondrial electron transport chain complex II expression in homogenates of control and IUGR placentas. After normalization to β-actin, the mean density of C samples was assigned an arbitrary value of 1. Subsequently, individual IUGR density values were expressed relative to this mean.. Values are given as means +S.E.M.; * $P < 0.05$ vs. control; unpaired Student's t test.

(b-d) Correlation between placental mTORC1 functional readouts $S6K^{T-389}$ (b), 4E-BP1 $^{T-37/46}$ (c) 4E-BP1 (d) and mitochondrial electron transport chain complex II expression. r= Pearson correlation coefficient, n= Control, n=19; IUGR, n=25.

Supplemental Figure 6. Correlation between placental mTORC1 functional readouts and the protein expression of mitochondrial ETC complex III. (a) Relative expression of mitochondrial electron transport chain complex III expression in homogenates of control and IUGR placentas. After normalization to β-actin, the mean density of C samples was assigned an arbitrary value of 1. Subsequently, individual IUGR density values were expressed relative to this mean.. Values are given as means + S.E.M.; * P < 0.05 vs. control; unpaired Student's t test. (b-d) Correlation between placental mTORC1 functional readouts $S6K^{T-389}$ (b), 4E-BP1 $^{T-37/46}$ (c) 4E-BP1 (d) and mitochondrial electron transport chain complex III expression. r= Pearson correlation coefficient, n= Control, n=19; IUGR, n=25.

Supplemental Figure 7. Correlation between placental mTORC1 functional readouts and the protein expression of mitochondrial ETC complex IV. (a) Relative expression of mitochondrial electron transport chain complex IV expression in homogenates of control and IUGR placentas. After normalization to β-actin, the mean density of C samples was assigned an arbitrary value of 1. Subsequently, individual IUGR density values were expressed relative to this mean. Values are given as means + S.E.M.; * P < 0.05 vs. control; unpaired Student's t test. (b-d) Correlation between placental mTORC1 functional readouts $S6KT^{-389}$ (b), 4E-BP1 $^{T-37/46}$ (c) 4E-BP1 (d) and mitochondrial electron transport chain complex IV expression. r= Pearson correlation coefficient, n= Control, n=19; IUGR, n=25.

Supplemental Figure 8. Correlation between placental mTORC1 functional readouts and the protein expression of mitochondrial ETC complex V. (a) Relative expression of

mitochondrial electron transport chain complex V expression in homogenates of control and IUGR placentas. After normalization to β-actin, the mean density of C samples was assigned an arbitrary value of 1. Subsequently, individual IUGR density values were expressed relative to this mean.. Values are given as means + S.E.M.; * $P < 0.05$ vs. control; unpaired Student's t test. (b-d) Correlation between placental mTORC1 functional readouts S6K^{T-389} (b), 4E-BP1 $^{T-37/46}$ (c) 4E-BP1 (d) and mitochondrial electron transport chain complex V expression. r= Pearson correlation coefficient, n= Control, n=19; IUGR, n=25.

Supplemental Figure 9. (a-d) Protein expression of raptor, rictor and DEPTOR in placental homogenates of control and IUGR group. Representative Western blots are shown. (b) Relative expression of raptor, rictor and DEPTOR expression in placental homogenates of control and IUGR. After normalization to β-actin, the mean density of C samples was assigned an arbitrary value of 1. Subsequently, individual IUGR density values were expressed relative to this mean. Values are given as means + S.E.M; *, $P < 0.05$ vs. control; unpaired Student's t test (Control, n=19; IUGR, n=25).

References:

1 Kanehisa, M., Furumichi, M., Tanabe, M., Sato, Y. & Morishima, K. KEGG: new perspectives on genomes, pathways, diseases and drugs. *Nucleic Acids Res* **45**, D353-D361 (2017).

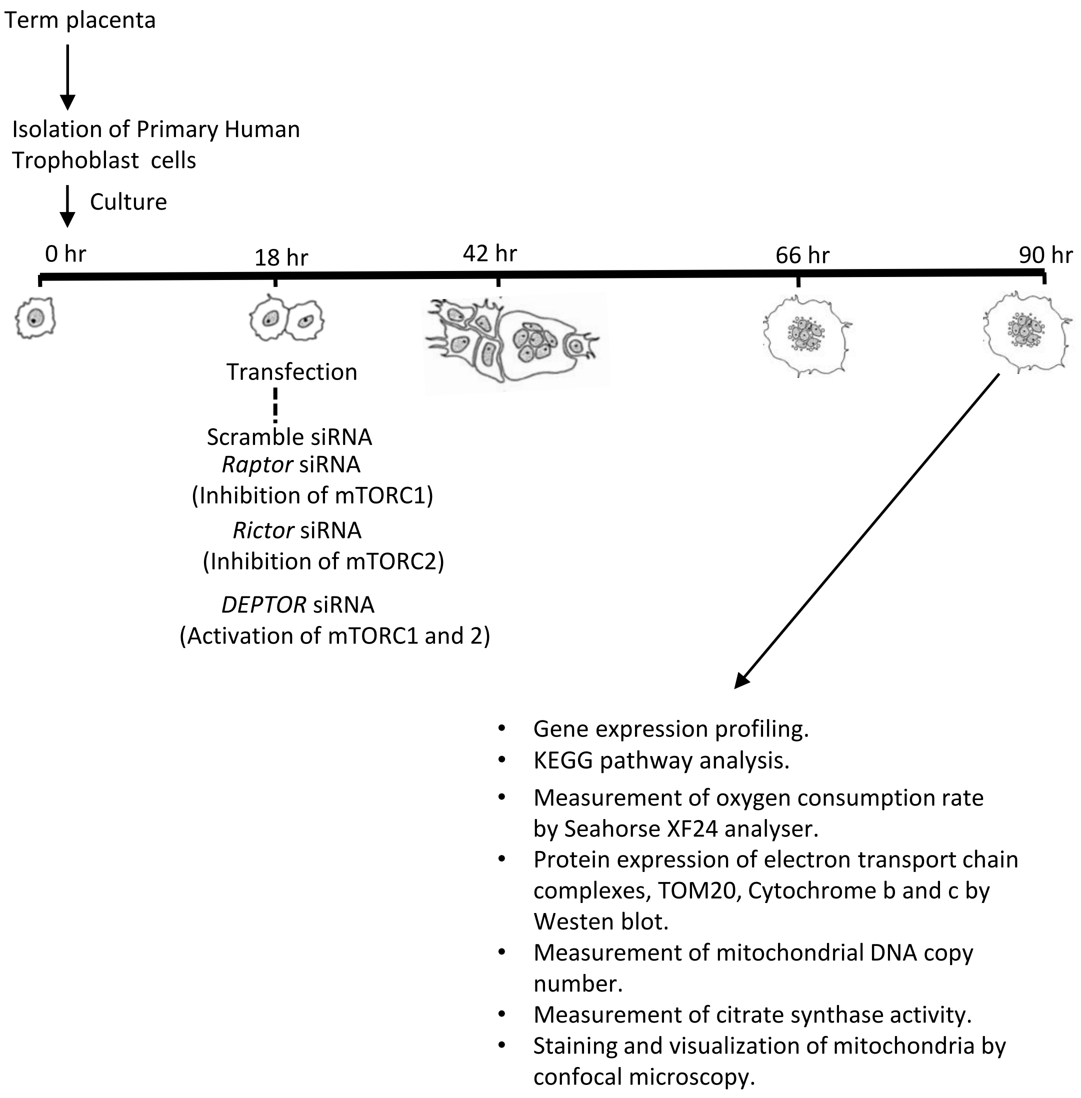

Supplementary Figure-1

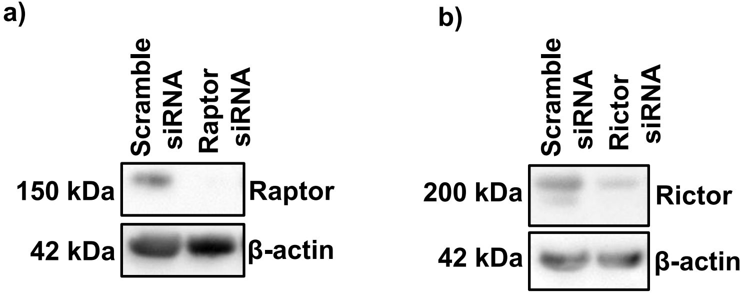

Supplemental Figure 2

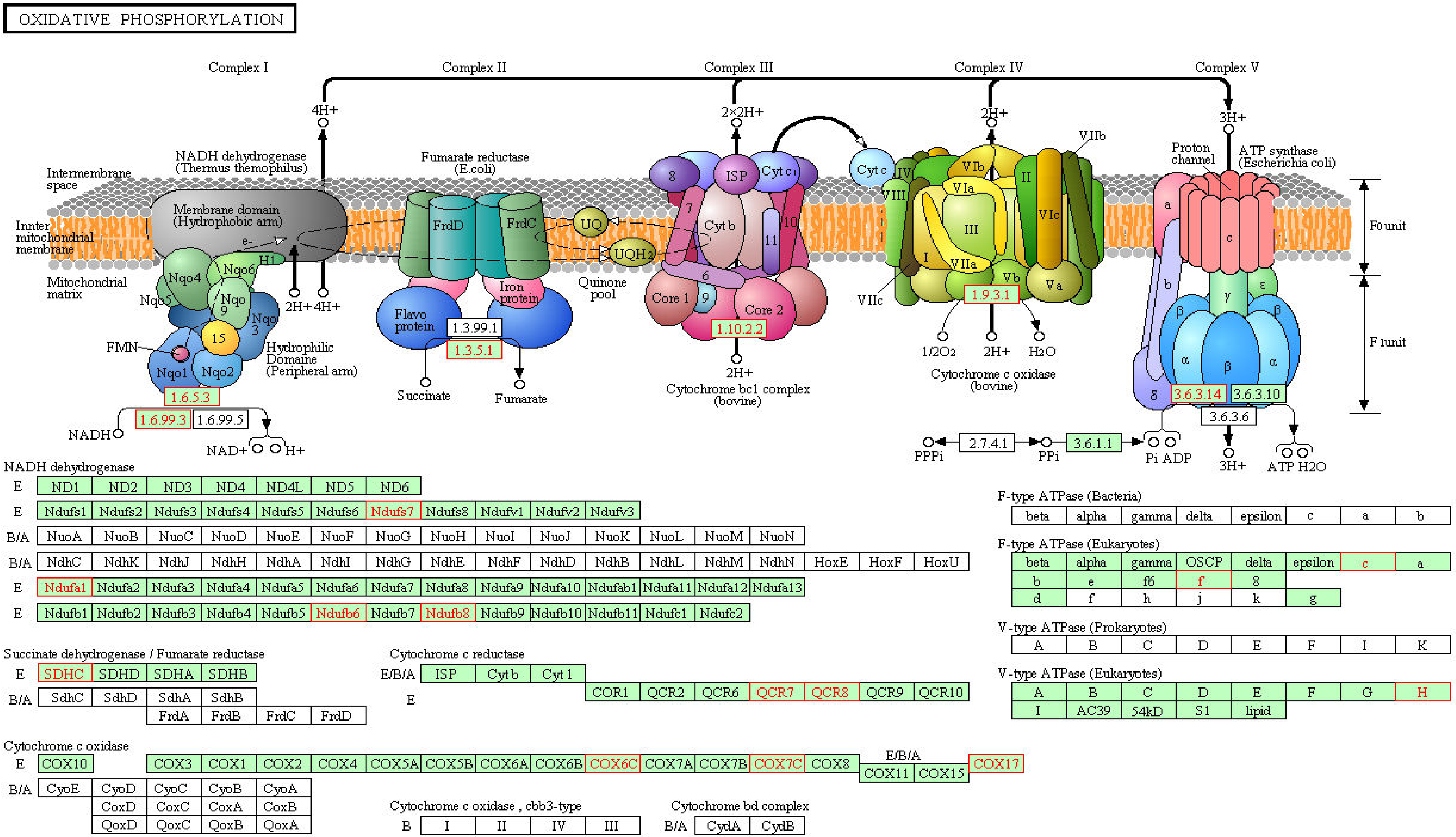

Supplemental Figure 3

a)

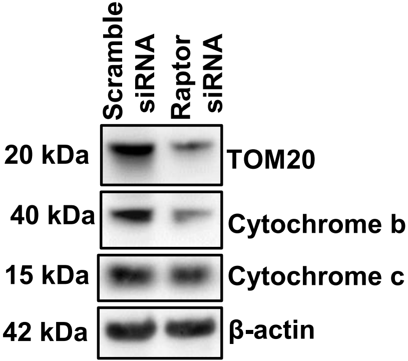

Supplemental Figure 4

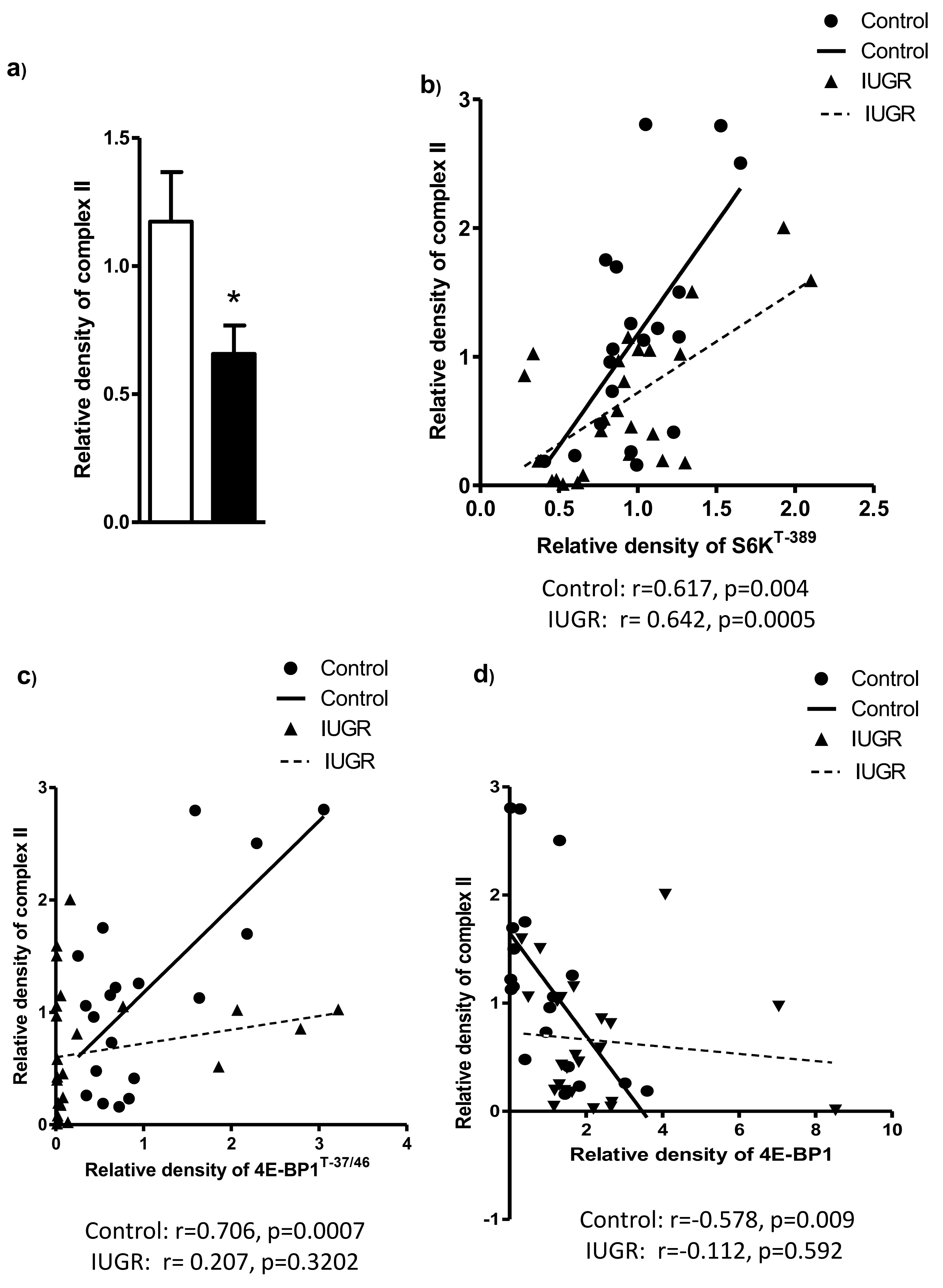

Supplemental Figure 5

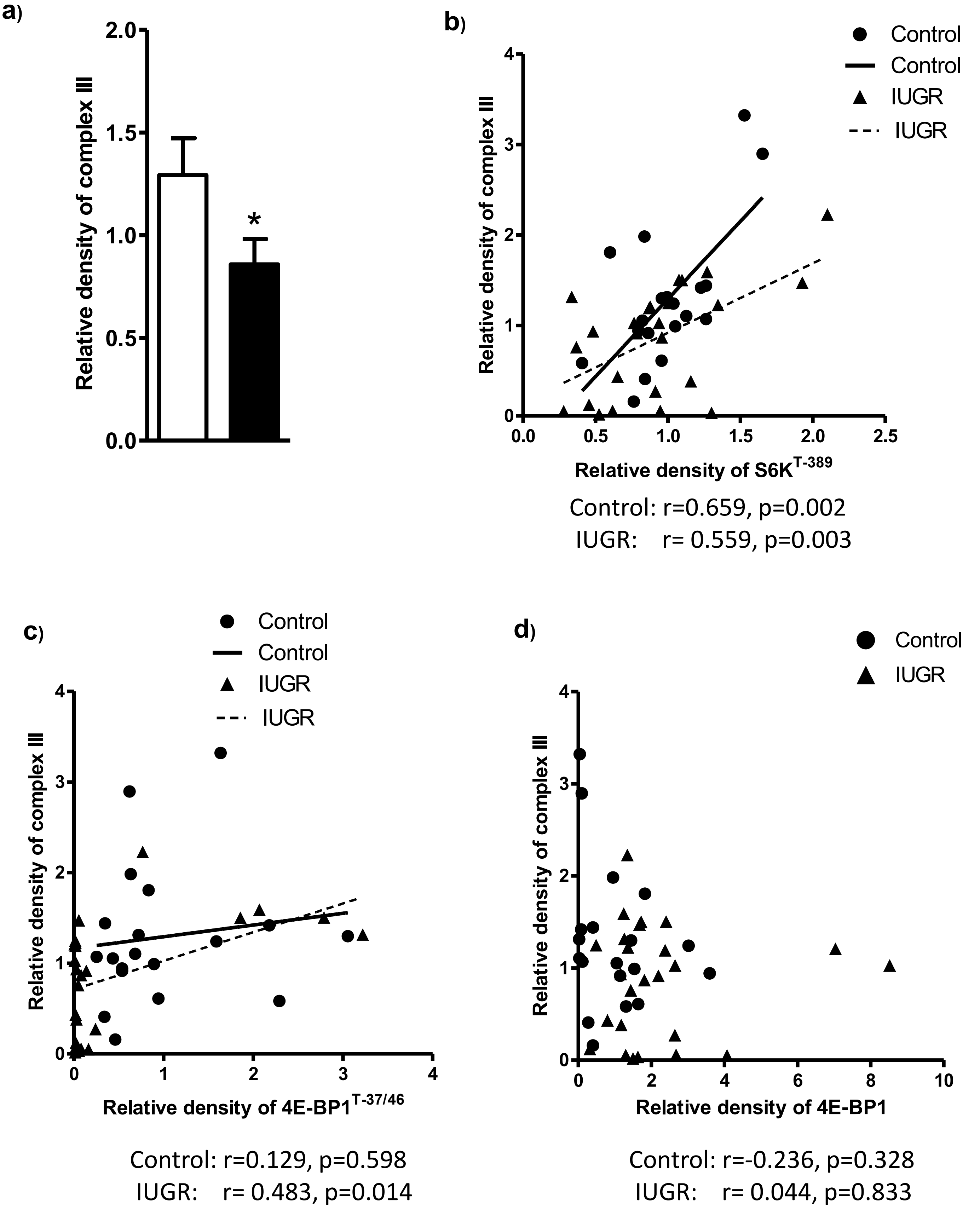

Supplemental Figure 6

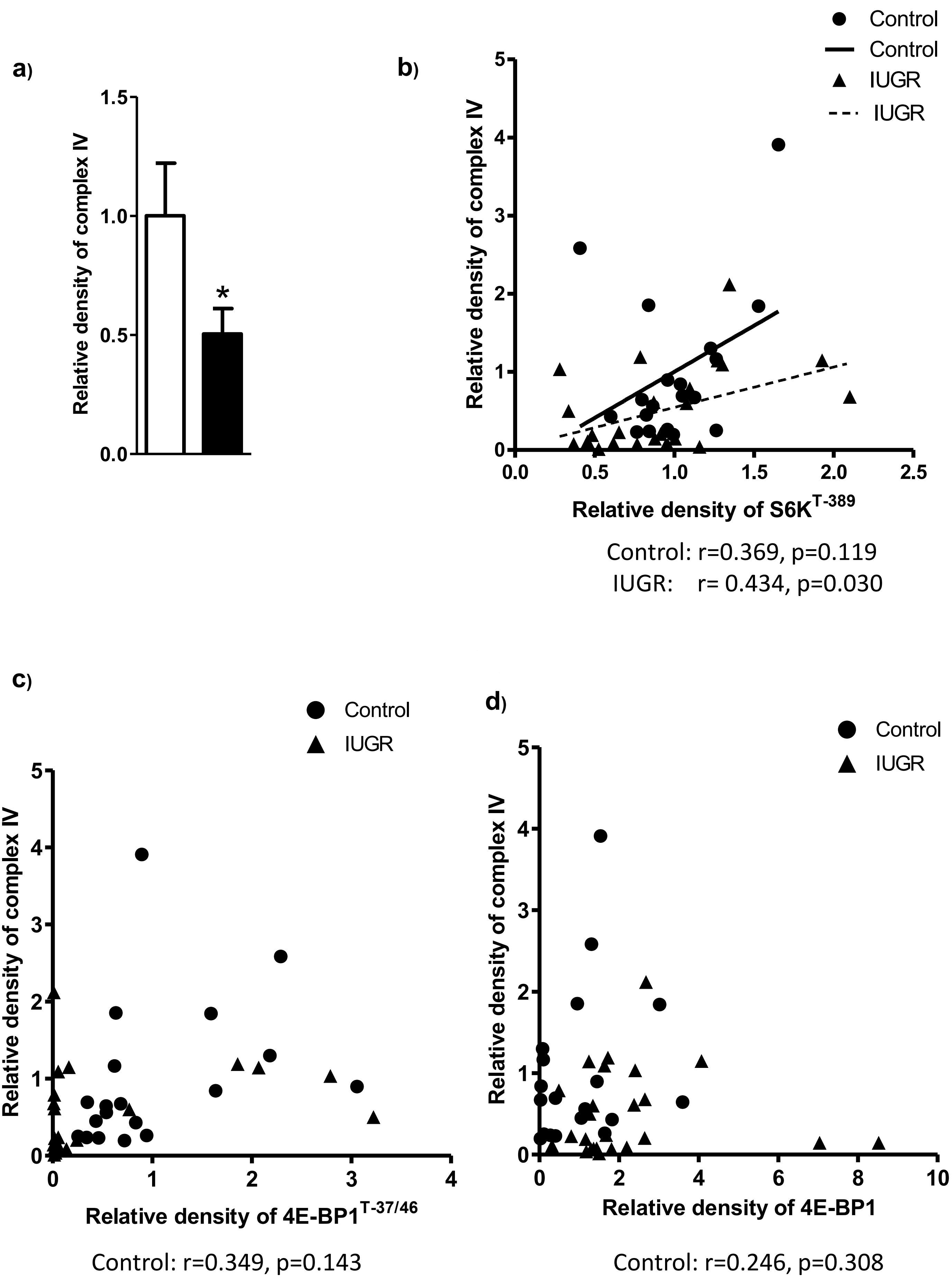

Control: r=0.369, p=0.119
IUGR: r= 0.434, p=0.030

Control: r=0.349, p=0.143
IUGR: r= 0.335, p=0.101

Control: r=0.246, p=0.308
IUGR: r= -0.005, p=0.978

Supplemental Figure 7

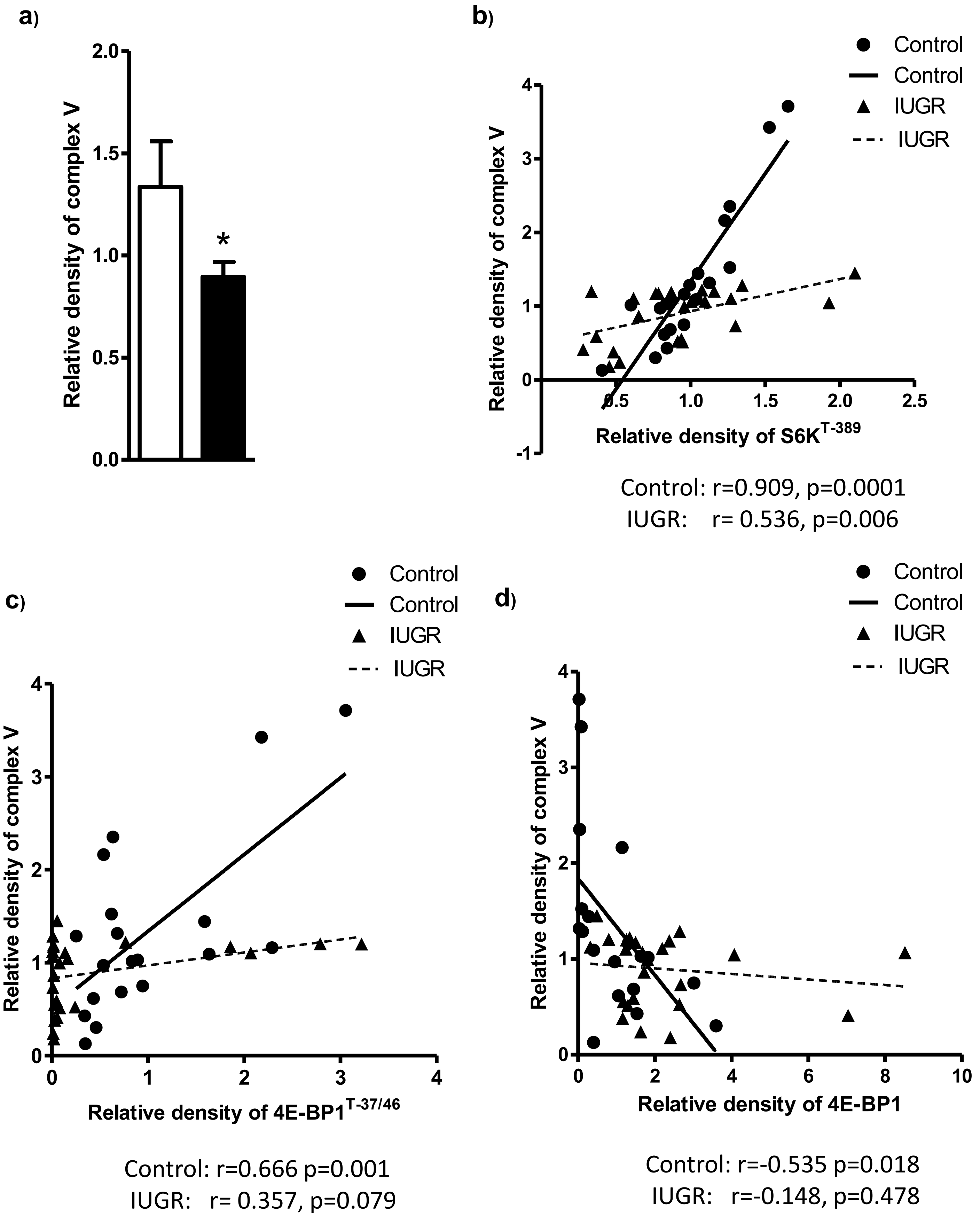

Supplemental Figure 8

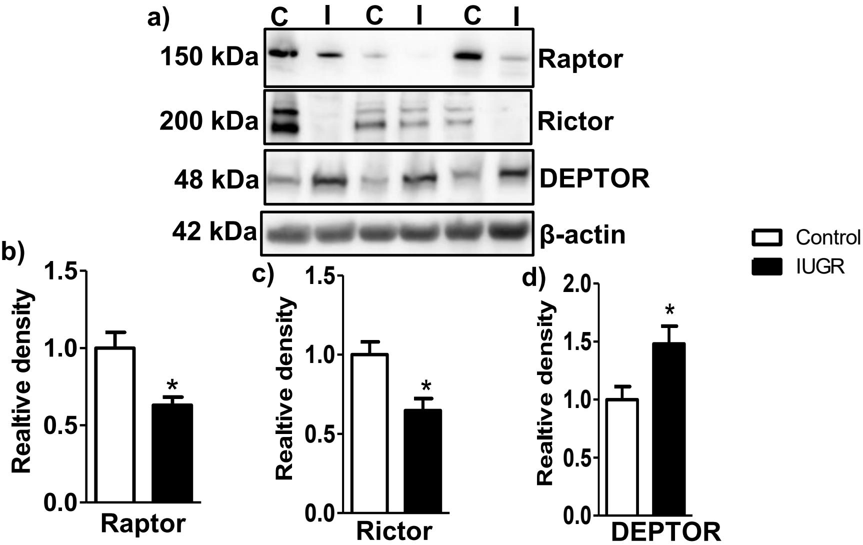

Supplemental Figure 9
